# Sequential binding of ezrin and moesin to L-selectin regulates monocyte protrusive behaviour during transendothelial migration

**DOI:** 10.1242/jcs.215541

**Published:** 2018-07-04

**Authors:** Angela Rey-Gallardo, Hannah Tomlins, Justin Joachim, Izajur Rahman, Phoebe Kitscha, Karen Frudd, Maddy Parsons, Aleksandar Ivetic

**Affiliations:** 1 School of Cardiovascular Medicine and Sciences, James Black Centre, BHF Centre of Research Excellence, 125 Coldharbour Lane, King's College London, London SE5 9NU, UK; 2 School of Basic & Medical Biosciences, Randall Division of Cell & Molecular Biophysics, New Hunt's House, London, SE1 1UL, UK

**Keywords:** FRET, FLIM, Ectodomain shedding, Polarity, Transmigration, Invasion

## Abstract

Leukocyte transendothelial migration (TEM) is absolutely fundamental to the inflammatory response, and involves initial pseudopod protrusion and subsequent polarised migration across inflamed endothelium. Ezrin/radixin/moesin (ERM) proteins are expressed in leukocytes and mediate cell shape changes and polarity. The spatio-temporal organisation of ERM proteins with their targets, and their individual contribution to protrusion during TEM, has never been explored. Here, we show that blocking binding of moesin to phosphatidylinositol 4,5-bisphosphate (PIP_2_) reduces its C-terminal phosphorylation during monocyte TEM, and that on–off cycling of ERM activity is essential for pseudopod protrusion into the subendothelial space. Reactivation of ERM proteins within transmigrated pseudopods re-establishes their binding to targets, such as L-selectin. Knockdown of ezrin, but not moesin, severely impaired the recruitment of monocytes to activated endothelial monolayers under flow, suggesting that this protein plays a unique role in the early recruitment process. Ezrin binds preferentially to L-selectin in resting cells and during early TEM. The moesin–L-selectin interaction increases within transmigrated pseudopods as TEM proceeds, facilitating localised L-selectin ectodomain shedding. In contrast, a non-cleavable L-selectin mutant binds selectively to ezrin, driving multi-pseudopodial extensions. Taken together, these results show that ezrin and moesin play mutually exclusive roles in modulating L-selectin signalling and shedding to control protrusion dynamics and polarity during monocyte TEM.

## Abstract (precis)

**Summary:** Ezrin and moesin co-ordinate binding to L-selectin in monocytes to, respectively, regulate pseudopod protrusion and ectodomain shedding during transendothelial migration.

## INTRODUCTION

An essential feature of acute inflammation is the migration of circulating innate immune cells, such as monocytes, towards damaged/infected tissue (Nourshargh and Alon, 2014; Schimmel et al., 2017). Following recruitment to activated endothelial monolayers, leukocytes undergo a series of dynamic changes in cell shape that are controlled by the coordinated actions of numerous cell adhesion molecules and underlying cytoskeletal proteins. For example, the ezrin/radixin/moesin (ERM) proteins link the plasma membrane and the underlying cortical actin-based cytoskeleton to mediate changes in cell shape (Fehon et al., 2010; Ivetic and Ridley, 2004a). Leukocytes express ezrin, moesin and negligible levels of radixin. ERM proteins can be structurally broken down into a band 4.1 ERM (FERM) N-terminal membrane-binding domain, a central α-helical domain and an acidic C-terminal actin-binding domain. ERM proteins shuttle dynamically between active and inactive states, the balance of which is usually tipped one way or the other by a diverse range of extracellular input signals. When inactive, ERM proteins adopt an auto-inhibited folded conformation, where the N- and C-termini interact to mask respective membrane- and F-actin-binding sites (Gary and Bretscher, 1995). Membrane binding is mediated principally through interaction of the FERM domain with phosphatidylinositol 4,5-bisphosphate (PIP_2_), which is enriched in the inner leaflet of the plasma membrane. PIP_2_ binding promotes the unfolding and activation of ERM proteins (Barret et al., 2000; Niggli et al., 1995). Threonine phosphorylation (residue 558 in moesin and 567 in ezrin) proximal to the actin-binding site acts to stabilise the PIP_2_-bound unfolded state (Pietromonaco et al., 1998; Simons et al., 1998). In addition to PIP_2_ binding, the FERM domain also interacts with the cytosolic domains of numerous transmembrane proteins (Hamada et al., 2003). By anchoring receptors to the underlying actin cortex, ERM proteins restrict lateral mobility and modulate clustering for signal transduction (Treanor et al., 2011). In this regard, ERM proteins play an essential role in modulating diverse signal transduction events. Despite their high level of similarity, different ERM protein members exhibit non-redundant roles in multiple cell types, including leukocytes (Ilani et al., 2007).

Leukocyte recruitment involves a series of increasingly adhesive interactions with cytokine-activated endothelial cells; this process can be broken down into unique observable cellular behaviours: tethering, rolling, slow rolling, firm adhesion and transendothelial migration (TEM). L-selectin is a type-I transmembrane cell adhesion molecule that mediates tethering and rolling, and is one of many surface receptors anchored to the cortical actin cytoskeleton by ERM protein binding (Ivetic, 2013, 2018; Ivetic et al., 2002; Ivetic and Ridley, 2004b). Recent evidence suggests L-selectin can regulate pseudopod protrusion and polarity in monocytes (Rzeniewicz et al., 2015). More specifically, L-selectin contributes to monocyte invasion during TEM. Upon encountering subendothelial ligands (such as biglycan), the pool of L-selectin within membranes of transmigrated pseudopods is clustered into distinct foci, which precedes localised ectodomain shedding. The biological role of L-selectin shedding within transmigrated pseudopods is to fine-tune the extent of cell protrusive behaviour once TEM is successfully completed. Pharmacological blockade of L-selectin shedding in primary human monocytes does not impede TEM per se, but dramatically alters front–back polarity and directional persistence in migration of fully transmigrated cells (Rzeniewicz et al., 2015). This is further supported by *in vivo* studies, where genetic blockade of L-selectin shedding dramatically impairs neutrophil interstitial chemotaxis towards ‘intermediary chemokines’ that bind CXCR2. These observations imply possible conserved mechanisms in the way L-selectin impacts on protrusive behaviour in neutrophils; however, this is currently speculative (Venturi et al., 2003). Although ERM proteins interact with the cytoplasmic tail of L-selectin, their contribution to regulating pseudopod protrusion during TEM has never been investigated. L-selectin is anchored to ERM protein-enriched microvilli and is rapidly cleaved by the sheddase ADAM17 within minutes of cell activation [e.g. with phorbol myristate acetate (PMA) or TNF]. Mutation of a membrane-proximal arginine residue at position 357 in the L-selectin tail to alanine (R357A) is sufficient to abrogate ERM protein binding altogether (Ivetič et al., 2004). R357A L-selectin anchors poorly to microvilli, which manifests in reduced leukocyte tethering efficiency under flow conditions. Intriguingly, R357A L-selectin can resist PMA-induced shedding; this implies that ERM proteins act as ‘pro-shedding factors’. Given that the interaction between L-selectin and ERM proteins supports microvillar anchoring for leukocyte tethering under flow, it seems contradictory for ERM protein binding to equally drive ectodomain shedding. A simple resolution to this paradox could be that ezrin and moesin possess mutually exclusive roles in regulating L-selectin function. Evidence from biochemical studies shows that moesin binds to the L-selectin tail following cell activation, whereas ezrin interacts with L-selectin under both resting (unchallenged) and cell-activating conditions (Ivetic et al., 2002). In this report, we show that ezrin and moesin indeed play unique roles in regulating leukocyte recruitment. Moreover, we expose a previously uncharacterised behaviour of ERM proteins: sequential binding to a common target to mediate mutually exclusive roles in regulating cell protrusive behaviour during TEM.

## RESULTS

### Regulation of ERM protein activity during TEM

**Fig. 1.**
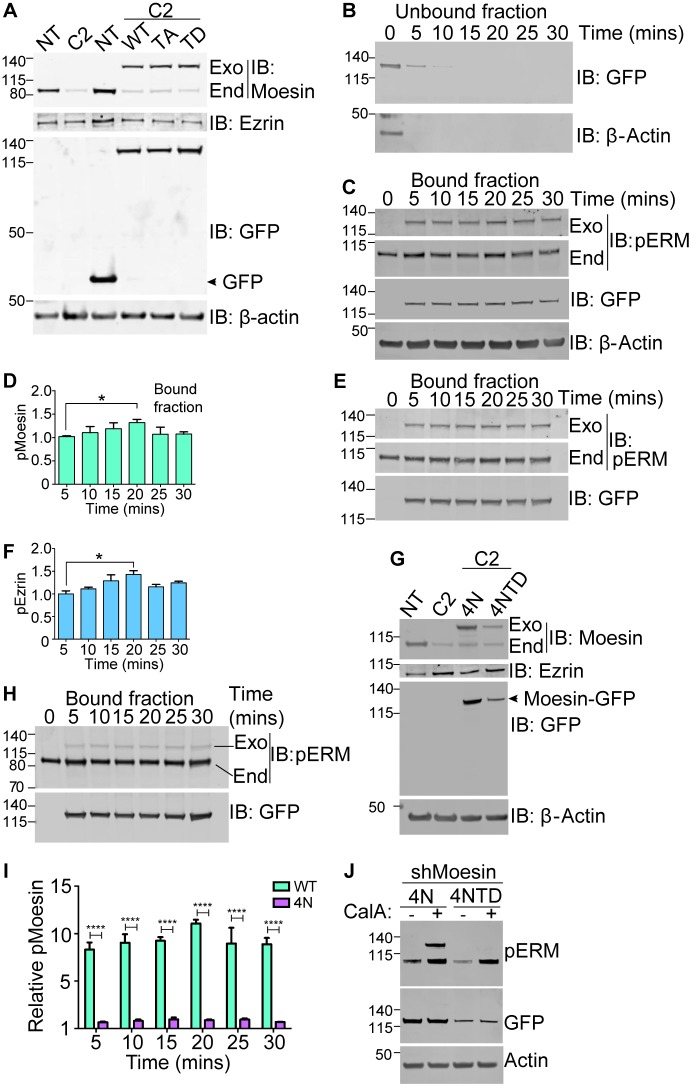
**Monitoring of ezrin and moesin activation during TEM in THP-1 cell lines reconstituted with shRNA-resistant ezrin–GFP and moesin–GFP.** (A) Reconstitution of WT, constitutively inactive (TA) or constitutively active (TD) mutant moesin–GFP into cells expressing clone number 2 (C2) shRNA. Non-specific targeting (NT) shRNA at a multiplicity of infection (MOI) of 10 was used as control in all experiments, and in relevant cases GFP alone was expressed in place of reconstituted moesin–GFP. Relative electrophoretic mobilities of endogenous (End) and exogenous (Exo) moesin are indicated on the immunoblot. Immunoblotting (IB) for ezrin revealed that no adverse effects were seen in endogenous ezrin expression in reconstituted cells, and β-actin bands represent loading control. (B–D) Monitoring C-terminal T558 phosphorylation levels in WT moesin–GFP-expressing cells during TEM (for details, see Materials and Methods). At each time point, whole-cell extracts were generated from THP-1 cells that were unbound (B) or bound (C) to the underlying TNF-activated HUVECs. Note that after 5 min, most THP-1 cells bound to the underlying endothelium. Bands representing phosphorylated moesin–GFP (Exo pERM on blots, pMoesin on graph) were normalised against GFP signals corresponding to the tag and results of three independent experiments are summarised in D. Note that the actin loading control in C suggests the majority of this signal is derived from HUVECs rather than THP-1 cells. (E,F) C-terminal phosphorylation of exogenously expressed ezrin–GFP (Exo pERM on blots, pEzrin on graph) was monitored in THP-1 cells expressing shRNA clone 5 as in C,D. Faithful knockdown and reconstitution of WT ezrin–GFP is depicted in Figs S1 and S2. Note that WT ezrin–GFP is resistant to clone 5 shRNA (target sequence in Materials and Methods section). (G) Generation and characterisation of THP-1 cells expressing clone 2 of moesin shRNA along with the reconstituted shRNA-resistant PIP_2_-binding mutant (4N) or phospho-mimic 4N (4NTD) moesin. (H,I) Immunoblots of whole-cell lysates of THP-1 cells expressing reconstituted 4N moesin–GFP, subjected to the TEM assay as in C–F. Quantification of immunoblots reveals that phosphorylation of 4N moesin–GFP (pMoesin) is dramatically reduced during TEM. Data are representative of three independent experiments. Relative T558 phosphorylation levels between 4N and WT moesin–GFP are directly compared in I. (J) Forced activation of 4N moesin–GFP with 25 nM of the phosphatase inhibitor calyculin A (CalA). Note that 4NTD moesin is not detectable with the anti-phospho-ERM antibody. shMoesin, C2 shRNA against moesin. Error bars represent s.d. *P<0.05, ****P<0.0001 (one-way ANOVA followed by Bonferroni's post-test).

To monitor the subcellular organisation of ERM proteins during TEM, the human monocyte-like cell line THP-1 was subjected to lentiviral transduction with short hairpin RNA (shRNA) to deplete endogenous levels of moesin (Fig. S1A–D). In each case, endogenous ezrin levels were not affected by this procedure (Fig. S1E). Thereafter, shRNA-resistant GFP-tagged wild-type (WT), constitutively inactive (TA) or constitutively active (TD) moesin was expressed in the cells to similar levels (Fig. 1A). Immunoblotting of C-terminal threonine phosphorylation is typically used to biochemically quantify ERM protein activation in cells (Ivetic and Ridley 2004a). Given that moesin–GFP is 28 kDa greater than endogenous moesin, we could cleanly investigate the phosphorylation status of leukocyte-derived moesin during TEM. THP-1 cells expressing WT moesin–GFP were added to TNF-activated human umbilical vein endothelial cell (HUVEC) monolayers (see Materials and Methods). The shift from unbound (suspended) cells to bound cells peaked at between 5 and 10 min (Fig. 1B,C). Whole-cell lysates were collected at different time points for western blotting. By 20 min, phospho-moesin–GFP increased modestly, but significantly (Fig. 1D). This outcome was mirrored in THP-1 cells expressing WT ezrin–GFP, reconstituted in ezrin-knockdown cells (Fig. 1E,F; Figs S1 and S2). These data suggest that both ezrin and moesin are broadly under similar levels of regulation in monocytes undergoing TEM. However, these results provide no understanding of their subcellular localisation during TEM. Numerous studies have shown that PIP_2_ binding of moesin precedes phosphorylation of ERM proteins (Ben-Aissa et al., 2012; Lubart et al., 2018). To address the impact of PIP_2_ binding on moesin activation during TEM, a series of lysine (K) to asparagine (N) mutations at positions 253, 254, 262 and 263 (K253N, K254N, K262N and K263N), which are known to be important for PIP_2_ binding (Barret et al., 2000), were engineered into the moesin–GFP FERM domain (denoted 4N) and stably reconstituted into cells lacking endogenous moesin. The 4N mutant was also engineered to harbour the TD mutation at position 558 (hereafter denoted 4NTD), which would incapacitate membrane binding of constitutively activated moesin (Fig. 1G). Interestingly, the 4N moesin–GFP mutant was poorly phosphorylated in THP-1 cells undergoing TEM (Fig. 1H,I). This result suggests that PIP_2_ binding is essential for C-terminal phosphorylation – both under resting conditions and during TEM. Furthermore, the Mander's overlap co-efficient (MOC) between the GFP tag signal and actin (staining with TRITC–phalloidin) was significantly reduced in 4N cells under resting conditions (Fig. S3), confirming that the 4N mutant could not localise to membranes in monocytes. Importantly, forced activation of the 4N mutant with 25 nM calyculin A led to robust C-terminal phosphorylation of the moesin–GFP (Fig. 1J); thus, the need for PIP_2_ binding can be bypassed by exposing cells to a potent antagonist of phosphatase activity against ERM proteins.

### C-terminal ERM phosphorylation regulates pseudopod formation during TEM

**Fig. 2.**
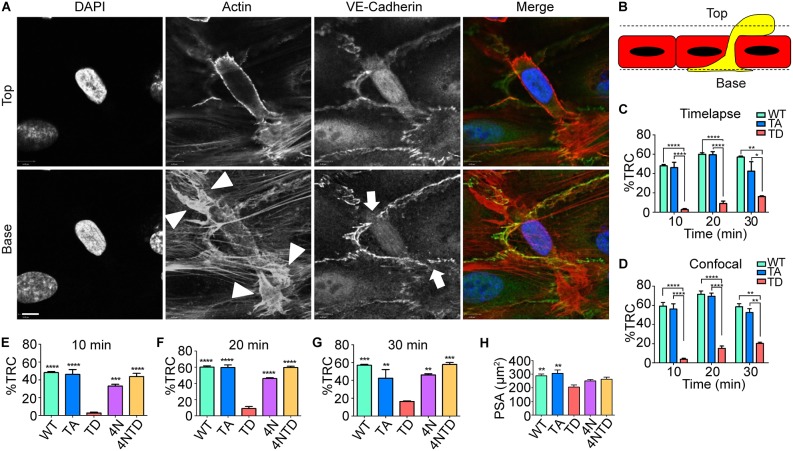
**Moesin dephosphorylation enables efficient pseudopod protrusion beneath activated endothelial monolayers.** (A,B) THP-1 cells were perfused continuously over TNF-activated endothelial monolayers for 15 min at 1.5 dynes per cm^2^ and subsequently fixed and prepared for point scanning confocal microscopy. Cells were stained with DAPI, phalloidin (actin) and anti-VE-cadherin antibody. Two optical sections were taken: above (top) and beneath (base) the endothelial monolayer. The relative locations of the ‘top’ and ‘base’ layers are depicted by dotted lines in the schematic in B. Arrowheads reveal positions of actin-rich protruding subendothelial pseudopods; arrows depict areas of discontinuous VE-cadherin expression, which is a hallmark of bona fide protrusion during TEM. Scale bars: 6 μm. (C,D) Quantification of TEM events in THP-1 cells expressing WT, TA or TD moesin expressed as a percentage of the total recruited cells (TRC) to the activated endothelial monolayer. Note that live-cell imaging (time-lapse, C) and confocal analysis (D) revealed very similar data. Fig. S4 provides visual examples of how cells were scored by time-lapse microscopy. (E–G) Data representing 4N and 4NTD cell lines overlaid with WT, TA and TD cells. Analysis was conducted every 10 min for a period of 30 min. (H) The pseudopodial spread area (PSA) within the subendothelial space of each cell line was quantified form the 30 min time point and expressed in µm^2^. Error bars represent s.e.m. *P<0.05, **P<0.01, ***P<0.001, ****P<0.0001 (compared with TD values; one-way ANOVA followed by Bonferroni's post-test).

We have recently reported that the THP-1 cell line is a useful model to study the molecular mechanisms governing leukocyte protrusion and polarity during TEM (Rzeniewicz et al., 2015). Perfusion of THP-1 cells over activated endothelial monolayers leads to their rapid recruitment and polarisation across intercellular endothelial junctions, generating a subendothelial (transmigrated) pseudopod and a non-transmigrated uropod (Fig. 2A,B). Imaging the spatio-temporal organisation of ERM proteins in uropods and pseudopods enables a clearer understanding of how protrusion and polarity during TEM is regulated at the molecular level. THP-1 cells expressing WT or mutant moesin–GFP were perfused over TNF-activated HUVECs for a period of 30 min, and protrusion dynamics were monitored at 10 min intervals. Evidence from both time lapse (dynamic) and confocal [paraformaldehyde (PFA)-fixed] microscopy revealed a significant reduction in the accumulation of constitutively active (TD) moesin–GFP within subendothelial pseudopods across all time points (Fig. 2C,D; Fig. S4). Cells expressing 4N moesin–GFP possessed fewer subendothelial pseudopods compared to WT cells (Fig. 2E–G). TD cells, however, produced the lowest number of subendothelial pseudopods, suggesting that inactivation of ERM proteins is an essential step towards pseudopod formation in TEM. Of the small proportion of TD-expressing cells that formed subendothelial pseudopods, the pseudopodial spread area (PSA) was significantly less compared to cells expressing WT or constitutively inactive (TA) moesin (Fig. 2H). Our data strongly suggest that inactivation of ERM proteins is an essential step in the establishment and maturation of subendothelial pseudopods during TEM. This conclusion was further ratified by using the 4NTD mutant cell line, which is constitutively active (open conformation) but unable to bind PIP_2_. Importantly, the 4NTD cell line established subendothelial pseudopods that were indistinguishable in frequency and size to those in WT cells, yet they differed radically to TD cells (Fig. 2E–G).

Given that L-selectin is a known ERM protein-binding partner that resides in both uropods and pseudopods of transmigrating monocytes, we next questioned whether L-selectin preferentially interacts with TD rather than TA moesin. THP-1 cell lines co-expressing WT L-selectin–GFP and either TA or TD moesin–RFP were perfused over TNF-activated HUVECs for 6 or 25 min, reflecting the early and late stages of TEM. Importantly, these two time points reflect when L-selectin shedding is minimal and maximal within transmigrated pseudopods (see Materials and Methods under ‘timed perfusion assays’) (Rzeniewicz et al., 2015).

**Fig. 3.**
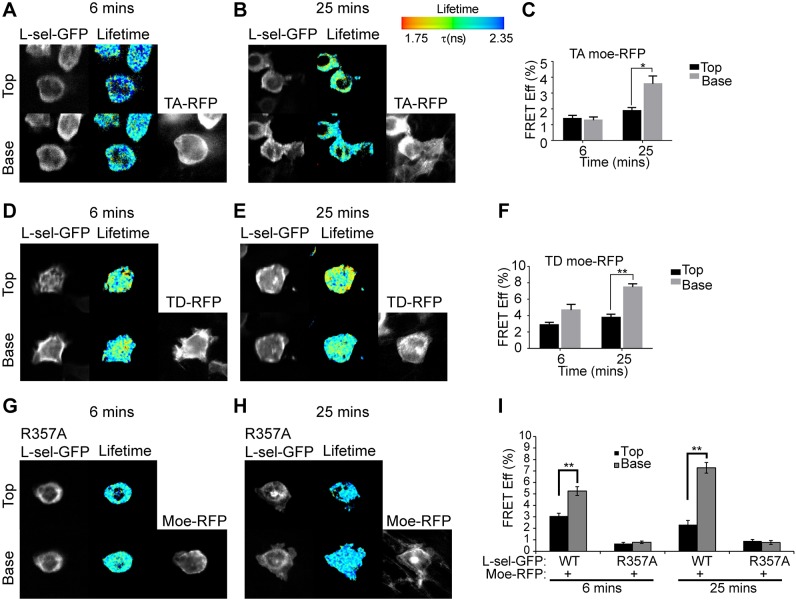
**T558 on moesin and R357 on L-selectin are essential amino acid residues for regulating the moesin–L-selectin interaction during TEM.** Cells expressing either TA (A–C) or TD (D–F) moesin–RFP (TA moe-RFP) with L-selectin–GFP (L-sel-GFP) were perfused over TNF-activated endothelial monolayers for either 6 or 25 min, which represent the early or late phases of TEM (and when ectodomain shedding of L-selectin shedding is, respectively, at a minimum and maximum within transmigrating pseudopods; Rzeniewicz et al., 2015). FRET efficiencies (FRET Eff) were measured by FLIM at two optical sections: above (top) and below (base) the HUVEC monolayer and representative images are shown (A,B,D,E). FRET efficiencies were quantified from three independent experiments, with at least 15 cells monitored on each day (C,F). (G–I) THP-1 cells co-expressing moesin–RFP and R357A L-selectin–GFP were perfused over TNF-activated endothelial cells for 15 min of continuous perfusion and quantified as in C and F. The lifetime of fluorescence is expressed in a pseudocolour scale from red (low lifetime with a very high probability of interaction) to blue (high lifetime with a very low probability of interaction). Note that RFP channel was taken in wide field and so could not be used to represent ‘top’ and ‘base’ images in this channel. Error bars represent s.e.m. *P<0.05, **P<0.01 (one-way ANOVA followed by Bonferroni's post-test).

Fluorescence lifetime imaging microscopy (FLIM) was used to quantify the fluorescence resonance energy transfer (FRET) efficiency between moesin–RFP and L-selectin–GFP in THP-1 cells captured during mid-TEM. Of the small amount of TD moesin entering transmigrated pseudopods, the FRET efficiency between TD moesin and L-selectin was significantly higher than with TA moesin and L-selectin (3.7% versus 7.8%, respectively; Fig. 3A–C and Fig. 3D–F). We have previously shown that interaction between L-selectin and ERM proteins is blocked by mutagenesis of arginine at position 357 into alanine (R357A) (Ivetič et al., 2004). Within our perfusion/transmigration assay, the R357A mutation completely blocked moesin–L-selectin interaction in both subcellular locations at both time points (Fig. 3G–I). Taken together, these results show that ERM protein cycling between active and inactive states is required for pseudopod formation and reconnection to transmembrane subendothelial targets, such as L-selectin.

### Mutually exclusive roles for ezrin and moesin during both recruitment and TEM of monocytes under flow conditions

**Fig. 4.**
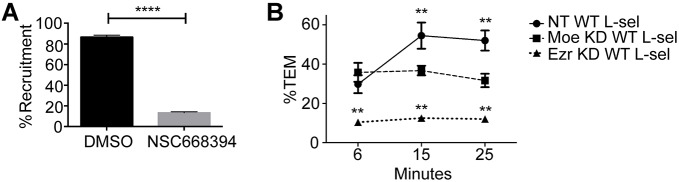
**Pharmacological or genetic blockade of ezrin function impacts on early phases of leukocyte recruitment.** (A) Primary human CD14^+^ monocytes were isolated from healthy donors on the day of the assay, pretreated for 10 min with either 10 µM of the ezrin inhibitor (NSC668394) or an equal volume of carrier (DMSO), and then perfused over TNF-activated endothelial monolayers at 1.5 dynes cm^2^ for a period of 20 min. Percentage recruitment was scored as the proportion of CD14^+^ cells stably interacting with the underlying endothelium, which included transmigrated cells. Rolling cells were excluded from the scoring, but made up the total percentage of recruited cells to 100%. (B) THP-1 cell lines stably expressing L-selectin–GFP (L-sel), but lacking endogenous ezrin or moesin expression by virtue of stable expression of shRNA clones for ezrin (Ezr KD, clone 5 or 6) or moesin (Moe KD, clone 2 or 3). Cells were perfused at 1.5 dynes/cm^2^ and analysed by time-lapse microscopy for a 25 min period. Cells were continually perfused over monolayers at a density of 0.5×10^6^ cells per ml. THP-1 cells expressing non-targeting shRNA (NT) along with L-selectin–GFP were used in control experiments. Data is expressed as the percentage of cells actively engaged in TEM over time. Significance is expressed. Error bars represent s.e.m. **P<0.01, ****P<0.0001 (compared with moesin-depleted cells in B; one-way ANOVA followed by Bonferroni's post-test).

Next, we sought to address the contribution of ERM proteins during primary human monocyte TEM. CD14^+^ monocytes constitute up to 85% of all circulating monocytes, and they also express L-selectin. Upon isolation, cells were pre-incubated with carrier alone (DMSO) or 10 µM of the ezrin inhibitor NSC668394. Previous studies have confirmed that NSC668394 blocks ezrin C-terminal threonine phosphorylation, ezrin–actin interaction and ezrin-dependent motility of osteosarcoma cells in culture (Bulut et al., 2012). Moreover, 10 µM NSC668394 was sufficient to block TEM of osteosarcoma cells across HUVEC monolayers under ‘static’ conditions (devoid of flow). Following drug treatment, CD14^+^ monocytes were continually perfused over TNF-activated HUVECs for 20 min and recruitment was scored as the number of cells that either adhered or transmigrated across the endothelial monolayer over this period (see Materials and Methods). Perfusion of DMSO-treated peripheral blood mononuclear cells (PBMCs) led to an average accumulation of 134 cells per field of view (FoV). Pre-treatment with 10 µM NSC668394 decreased recruitment by 85%, leaving 20 cells per FoV (Fig. 4A). These results imply that monocyte recruitment to TNF-activated HUVECs is sensitive to NSC668394, and that ezrin (or, more broadly, ERM protein activity) is likely to play an essential part in this mechanism. As moesin and ezrin are structurally conserved within regions targeted by NSC668394, we could not formally exclude the possibility that moesin is also a target in this inhibitor study. Furthermore, HUVEC-derived ERM proteins, which also contribute to leukocyte recruitment and TEM, are likely to be impacted by NSC668394.

Based on the inhibitor study, we were motivated to express WT L-selectin–GFP in THP-1 monocytes that were systematically depleted of cellular levels of ezrin or moesin protein by means of shRNA in order to determine their relative contribution to TEM. THP-1 monocytes do not normally express L-selectin, providing a useful cellular model to cleanly investigate the contribution of GFP-tagged WT or mutant L-selectin to TEM. Importantly, we have shown that GFP tagging of the C-terminal tail of L-selectin does not interfere with: (1) its binding partners (e.g. calmodulin and ERM proteins), (2) ectodomain shedding, and (3) tethering/rolling on immobilised sialyl Lewis^x^ (the archetypal glycan to which all selectins can bind) (Rzeniewicz et al., 2015). Two separate shRNA-targeted cell lines were continually perfused over TNF-activated HUVECs for 25 min. The number of cells actively engaged in TEM was calculated as a percentage of the total cells recruited to the endothelium from flow after 6, 15 and 25 min (see Materials and Methods). Binding of control cells expressing non-targeting shRNA (NT cells) to HUVECs under flow reached saturation by 15 min. Ezrin-depleted cells bound the least favourably to TNF-activated HUVECs across all time points, peaking to only 10% of cells actively undergoing TEM (Fig. 4B). As progression through the multi-step adhesion cascade relies on successful execution of preceding steps, these data strongly suggest ezrin contributes to early stages of the adhesion cascade (possibly in tethering/rolling). Interestingly, depletion of moesin did not impact on initial recruitment from flow, but the percentage of cells actively engaged in TEM reached saturation by 6 min (Fig. 4B). Taken together, and in agreement with our ezrin inhibition data (Fig. 4A), ezrin strongly supports the early phases of the adhesion cascade, whereas moesin is likely to support later stages of the adhesion cascade, for example, TEM. Given that numerous leukocyte-derived cell adhesion molecules interact with ERM proteins [PSGL-1, ICAM-3, CD43, CD44 and PECAM-1 to name a few (Alonso-Lebrero et al., 2000; Gamulescu et al., 2003; Serrador et al., 1997, 1998; Tsukita et al., 1994)], it is impossible to attribute the ezrin or moesin knockdown phenotype exclusively to L-selectin–ERM protein interaction. Moreover, resolving the complex phenotype underlying the difference in recruitment between NT cells and moesin knockdown cells was beyond the scope of this work.

### Sequential binding of ezrin and moesin to L-selectin during TEM

**Fig. 5.**
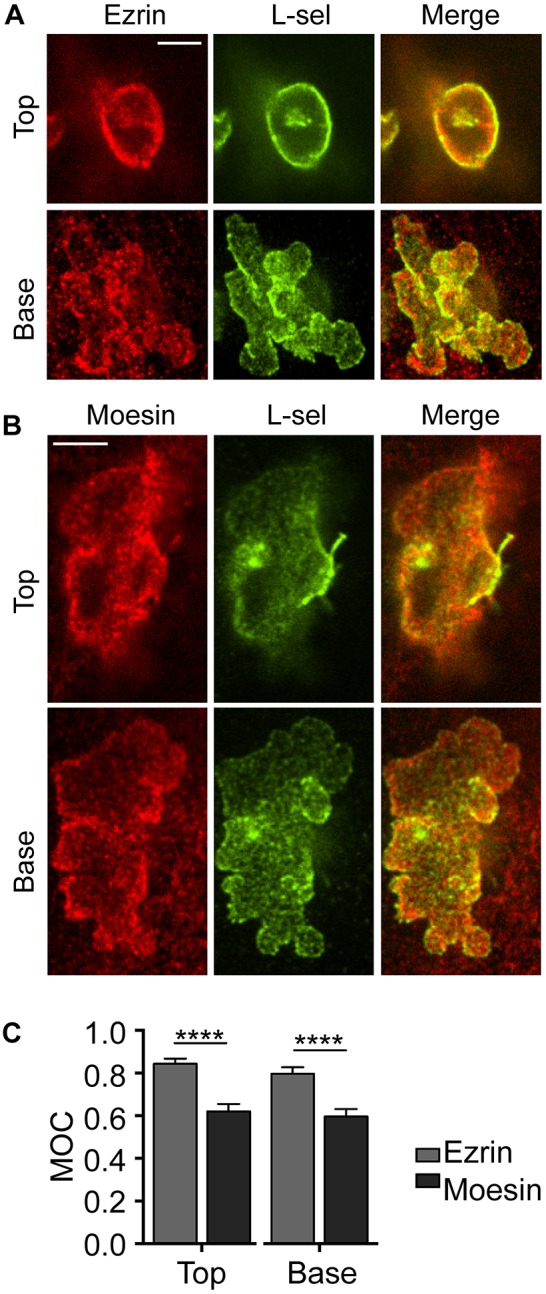
**Subcellular distribution of L-selectin with ezrin or moesin in primary human CD14^+^ monocytes during mid-TEM.** (A,B) Primary human CD14^+^ monocytes were perfused over TNF-activated endothelial monolayers for 3–6 min at a density of 1×10^6^ cells per ml so that the majority of cells could be captured in mid-TEM (see Movie 1). Samples were fixed immediately in 4% PFA and subsequently prepared for spinning disc confocal microscopy (see Materials and Methods). Anti-ezrin (A) and anti-moesin (B) antibodies were detected using Alexa Fluor 568 secondary antibodies and anti-L-selectin (L-sel; LAM1-14) was detected with Alexa 488 secondary antibody. Stills were taken from a wider imaging field (see Figs 5 and 6). Scale bars: 5 µm. Anti-PECAM-1 antibody and phalloidin 633 were used to validate bona fide TEM (see Figs 5 and 6). ‘Top’ and ‘base’ images represent optical sections of non-transmigrated and transmigrated regions, respectively. (C) Mander's overlap co-efficiency (MOC) between L-selectin and one ERM member was quantified from at least 45 cells over three independent experiments. Error bars represent s.e.m. ****P<0.0001 (two-tailed unpaired Student's *t*-test).

To determine whether L-selectin interacts preferentially with ezrin or moesin during TEM, primary human CD14+ monocytes were isolated from whole blood and perfused over TNF-activated HUVECs. Cells were subsequently fixed in 4% paraformaldehyde (PFA) following 3-6 min of perfusion, which captured the majority of monocytes in mid-TEM (see Movie 1). Each specimen was processed for immunofluorescence staining of actin, PECAM-1 (to ensure bona fide breach of intercellular junctions), L-selectin and ezrin or moesin. Collectively, these signals would provide strong evidence for bona fide TEM prior to monitoring the subcellular distribution of L-selectin with either ezrin or moesin. The MOC was calculated and used to quantify the subcellular distribution of L-selectin and ERM proteins within pseudopods and uropods (Fig. 5A,B; Figs S5 and S6). The MOC between L-selectin and ezrin was significantly higher (>26%) than that between L-selectin and moesin in both uropods and pseudopods of CD14^+^ monocytes (Fig. 5C). Although the MOC provides a quantitative measure of overlap between signals corresponding to L-selectin and moesin or ezrin, it does not indicate whether there is direct interaction between these proteins. Moreover, immunofluorescence staining for ‘total’ moesin or ezrin does not discriminate between active or inactive forms.

**Fig. 6.**
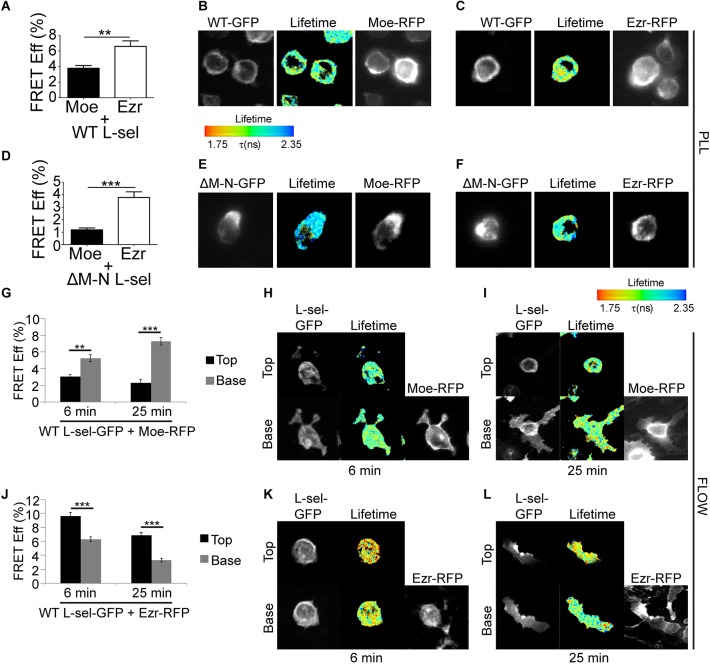
**Ezrin preferentially interacts with L-selectin in non-transmigrated uropods, whereas moesin interacts with L-selectin in transmigrated subendothelial pseudopods.** (A–F) THP-1 cells co-expressing either ezrin–RFP (Ezr-RFP) or moesin–RFP (Moe-RFP) (in ezrin- or moesin-knockdown cells, respectively), and WT or non-cleavable (ΔM-N) L-selectin–GFP, were resuspended into neat RPMI and seeded onto poly-L-lysine (PLL)-coated coverslips for 10 min at 37°C and subsequently fixed for FRET measurements by FLIM (see Materials and Methods). Representative images of each cell line, showing individual fluorescence channels and lifetime profiles, are provided. The lifetime of fluorescence is expressed in a pseudocolour scale from red (low lifetime with a very high probability of interaction) to blue (high lifetime with a very low probability of interaction). (G–L) THP-1 cells expressing WT L-selectin–GFP and ezrin–RFP or moesin–RFP (in ezrin- or moesin-knockdown cells, respectively) were perfused over TNF-activated HUVECs and fixed in 4% PFA at 6 min or 25 min to represent early and late phases of TEM, respectively (see ‘timed perfusions’ in the Materials and Methods). FLIM measurements were taken from two optical sections: above (‘Top’) and below (‘Base’) the endothelium. FRET efficiencies (FRET Eff) were derived from measurements of at least 45 cells from three independent experiments. Individual fluorescence channels and lifetime image are provided for each cell line. Note that RFP channel was taken in wide field and so could not be used to represent ‘Top’ and ‘Base’ images in this channel. Error bars represent s.e.m. **P<0.01, ***P<0.001 (two-tailed unpaired Student's *t*-test).

**Fig. 7.**
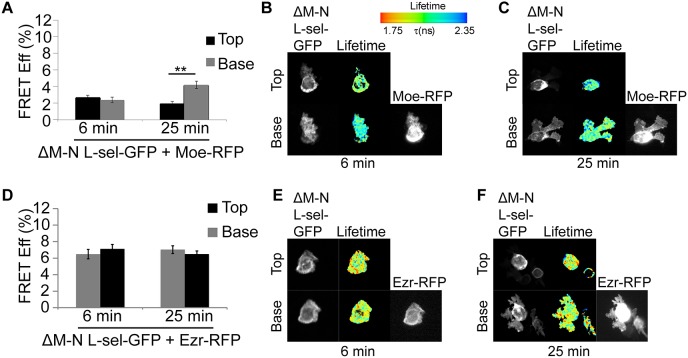
**Blocking shedding secures ezrin interaction exclusively with the L-selectin tail within transmigrating pseudopods.** THP-1 cells co-expressing ΔM-N L-selectin–GFP (L-sel-GFP) with either moesin–RFP (Moe-RFP) (A–C) or ezrin–RFP (Ezr-RFP) (D–F) (in moesin- or ezrin-knockdown cells, respectively) were perfused over TNF-activated HUVECs and fixed during the early (6 min) and late phases (25 min) of TEM. Measurements and analysis was conducted as in Fig. 6G–L. Note that RFP channel was taken in wide field and so could not be used to represent ‘top’ and ‘base’ images in this channel. Error bars represent s.e.m. **P<0.01 (two-tailed unpaired Student's *t*-test).

To monitor the direct interaction of L-selectin with either ezrin or moesin during TEM, THP-1 cells were engineered to co-express one shRNA-resistant ERM member tagged to RFP and WT L-selectin-GFP. Using FRET/FLIM techniques provides an excellent means to quantify the extent of interaction between L-selectin and ERM proteins within uropods and pseudopods of cells captured in mid-TEM. We recently reported that ectodomain shedding of L-selectin is activated within transmigrating pseudopods of CD14^+^ primary monocytes and THP-1 cell lines stably expressing WT L-selectin–GFP (Rzeniewicz et al., 2015). A non-cleavable mutant of L-selectin, called ΔM-N, was therefore included in the study to assess the impact of blocking L-selectin ectodomain shedding on its binding to ERM proteins (see Materials and Methods). In unchallenged cells, the FRET efficiency between WT L-selectin and ezrin was 1.7-fold higher than with moesin (Fig. 6A–C). Moreover, ezrin binding to ΔM-N L-selectin was 3.2-fold higher than with moesin (Fig. 6D–F). These results agree with the MOC data obtained from primary CD14^+^ monocytes showing preferential overlap between ezrin and L-selectin fluorescence signals (Fig. 5). Next, timed perfusion assays were conducted over TNF-activated endothelial monolayers at 6 min and 25 min, to reflect the early and late stages of TEM (Rzeniewicz et al., 2015) Importantly, these two time points reflect when L-selectin shedding is minimal and maximal within transmigrated pseudopods. The FRET efficiencies reflecting the interaction between moesin and WT L-selectin was consistently low within uropods at both time points: 3.1% and 2.1% at 6 and 25 min, respectively (Fig. 6G–I). However, the FRET efficiency corresponding to interaction between moesin and WT L-selectin in transmigrating pseudopods increased further from 5.3% to 7.3% by 25 min (Fig. 6G–I). Importantly, an inverse correlation was seen with the interaction between ezrin and WT L-selectin; there was consistently high FRET efficiencies within uropods (9.6% at 6 min to 6.8% at 25 min), but a progressive decrease within transmigrating pseudopods (6.3% at 6 min to 3.3% at 25 min; Fig. 6J–L). Of note, the pronounced moesin–L-selectin interaction in pseudopods at 25 min coincides with when ectodomain shedding of L-selectin is reportedly maximal in THP-1 cells (Rzeniewicz et al., 2015). This relationship is further supported by FRET efficiency measurements taken in cells expressing moesin and ΔM-N L-selectin, which was consistently low in both uropods (2.7% at 6 min and 2.0% at 25 min) and pseudopods (2.4% at 6 min and 4.2% at 25 min) (Fig. 7A–C). In stark contrast, the ezrin–ΔM-N interaction remained consistently high in uropods (6.5% at 6 min and 7.0% at 25 min) and pseudopods (7.1% at 6 min and 6.5% at 25 min) (Fig. 7D–F). Taken together, these results show that ezrin–L-selectin interaction dominates during early recruitment and TEM (e.g. during invasion). In contrast, moesin–L-selectin interaction is specifically increased within transmigrating pseudopods to mediate ectodomain shedding.

### The ezrin–L-selectin interaction drives multiple pseudopods in cells expressing ΔM-N L-selectin

**Fig. 8.**
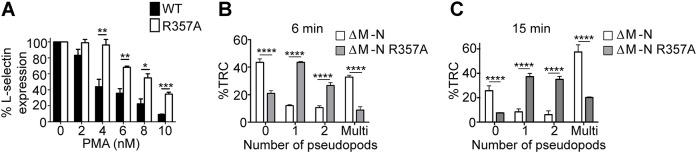
**Abrogating L-selectin–ERM protein interaction significantly reduces PMA-induced shedding and subendothelial protrusions in cells expressing** **ΔM-N L-selectin** **during TEM.** (A) THP-1 cells expressing WT or R357A L-selectin–GFP were challenged with increasing doses of PMA for 30 min at 37°C (see Materials and Methods). Cells were labelled with DREG56 (anti-L-selectin antibody) and surface levels of L-selectin analysed by flow cytometry and are presented as the percentage relative to that in untreated cells (0 nM, set at 100%). (B,C) THP-1 cells expressing either ΔM-N or ΔM-N R357A L-selectin were perfused over TNF-activated HUVECs and fluorescence time-lapse microscopy was used to measure the proportion of total recruited cells (%TRC) with the indicated number of subendothelial pseudopods projecting beneath the endothelium. Cells were scored for having zero, one, two or more than three (multi) protrusions at the 6 min and 15 min time points. Movies 2–4 represent the three cellular morphologies that were scored. A total of at least 300 cells per group were scored across three independent flow experiments. Error bars represent s.e.m. *P<0.05, **P<0.01, ***P<0.001, ****P<0.0001 (two-tailed unpaired Student's *t*-test)

Blocking the shedding of L-selectin in primary human CD14^+^ monocytes and THP-1 cells leads to defective front–back polarity through the production of excess and unstable pseudopodial extensions (Rzeniewicz et al., 2015). Given the strong propensity for ezrin, rather than moesin, to interact with non-cleavable ΔM-N L-selectin, it is likely that ezrin is responsible for driving the multi-pseudopod phenotype. Although systematic depletion of ezrin and moesin in THP-1 cells can broadly address their impact on recruitment (Fig. 4B), assigning a causal relationship between ezrin–L-selectin interaction and cell protrusion during TEM via this experimental approach is impossible. For example, other ezrin targets necessary for events that precede TEM could be affected and confuse interpretation. Therefore, to test a causal relationship between the ezrin–ΔM-N L-selectin interaction and the multi-protrusion phenotype, cells were engineered to express ΔM-N L-selectin containing either a WT cytoplasmic tail sequence or a single amino acid swap mutation that abrogates ERM protein binding altogether (denoted ΔM-N R357A). We have previously shown biochemically that the R357A mutation blocks binding to both ezrin and moesin (Ivetič et al., 2004). We found that THP-1 cells expressing R357A L-selectin–GFP phenocopied the resistance to PMA-induced shedding reported previously in 300.19 pre-B cells expressing untagged R357A L-selectin (Fig. 8A). Moreover, FLIM revealed negligible FRET efficiencies between moesin–RFP and R357A L-selectin–GFP in cells undergoing TEM (Fig. 3G–I). Perfusion of ΔM-N L-selectin cells over TNF-activated endothelial monolayers presented the typical multi-protrusion phenotype that was previously reported (see Movies 2–4). However, a significant reduction in the number of multiple protrusions was observed in cells expressing ΔM-N R357A L-selectin–GFP (Fig. 8B). Taken together, based on observations in Fig. 7D, our data strongly imply that the ezrin–ΔM-N L-selectin interaction is directly involved in driving the multi-protrusion phenotype.

## DISCUSSION

Here, we show that ezrin and moesin regulate unique aspects of L-selectin, which in turn impact on monocyte protrusive behaviour during TEM. We have previously shown that ectodomain shedding of L-selectin occurs specifically within transmigrating subendothelial pseudopods (Rzeniewicz et al., 2015). A switch from ezrin to moesin binding appears to be necessary in fine-tuning the invasive potential of L-selectin within transmigrating pseudopods. It seems that ectodomain shedding abruptly blocks signalling downstream from L-selectin. We speculate that during TEM, the pool of L-selectin within transmigrated pseudopods is corralled into clusters by moesin before it undergoes ectodomain shedding. We already know from previous work that ΔM-N L-selectin is never seen to cluster within transmigrating pseudopods of monocytes (Rzeniewicz et al., 2015). It is currently not clear whether the cleaved membrane-retained product of L-selectin continues to interact with moesin. If so, it would be interesting to determine whether the cleaved L-selectin–moesin complex provides any contribution to chemotaxis or in the establishment of front–back polarity of fully transmigrated cells. Our hypothetical view is supported by the finding that the non-cleavable mutant ΔM-N L-selectin preferentially interacts with ezrin and drives the excessive pseudopod protrusion phenotype. Moreover, this phenotype is copied in primary human CD14^+^ monocytes where L-selectin shedding has been blocked (Rzeniewicz et al., 2015). Importantly, expression of ΔM-N R357A L-selectin, which cannot bind to ERM proteins, reverses the multi-protrusion phenotype. Given that ezrin binds preferentially to ΔM-N L-selectin (Fig. 7D–F), it is highly likely that the ezrin–ΔM-N L-selectin interaction is driving the multi-protrusion phenotype. A key tyrosine residue within ezrin, Y353, which is absent in moesin, is a known target for c-Src. Phosphorylation of ezrin on Y353 in transformed epithelial cells leads to binding of the p85 regulatory subunit of PI3K (Gautreau et al., 1999). Indeed, the colocalisation of p85 and ezrin in T-cell receptor activated cells (Shaffer et al., 2009) suggests these proteins can interact in immune system cells. It is tempting to speculate that phosphorylation of Y353 in ezrin could lead to localised recruitment of p85 and the subsequent PI3K activation that is necessary for pseudopod extension during TEM (Fig. S7). Furthermore, this would explain why sustained interaction between L-selectin and ezrin, like that seen with ΔM-N L-selectin (Fig. 7D–F), would lead to less shedding and drive the multi-pseudopod phenotype. Although the L-selectin–ezrin interaction is driving protrusion, the molecular mechanism through which this is achieved will be an important focus for future studies. Clustering of L-selectin by means of physiological ligand or monoclonal antibody can activate β1 and β2 integrins in multiple cell types (Giblin et al., 1997; Green et al., 2004; Hwang et al., 1996; Morikis et al., 2017), upregulate chemokine receptor expression (Ding et al., 2003; Duchesneau et al., 2007) and increase T-cell responsiveness to chemokines (Subramanian et al., 2012). Collectively, all of these attributes could contribute to increased protrusive behaviour. A consequence of the multi-protrusion behaviour in response to blocking L-selectin shedding is that monocytes lose front–back polarity altogether. The establishment of front–back polarity in fully transmigrated monocytes is likely to be essential for their chemotaxis towards sites of injury/infection, although this will require *in vivo* validation.

It is important to note that total protein levels of moesin exceeds that of ezrin in immune cells, yet the highest MOC between L-selectin and ERM proteins in this study is consistently between ezrin and L-selectin (Fig. 5). The LAM1-14 monoclonal antibody used in this study recognises the extracellular domain of L-selectin above the cleavage site, which means that only cell-associated full-length, and not cleaved L-selectin, can be detected. The consistently lower MOC between L-selectin and moesin is highly suggestive of moesin acting as the pro-shedding factor, which is supported by the FLIM/FRET data in THP-1 cells. Our findings satisfy questions that were raised when the biochemical interaction between L-selectin and ERM proteins was first identified and characterised (Ivetic et al., 2002). The retention of ezrin, but not moesin, on L-selectin tail affinity columns was observed after passing extracts derived from unchallenged naive T-cells. However, the more abundant moesin was enriched on L-selectin affinity columns only when extracts were derived from PMA-stimulated T-cells (PMA is a potent agonist of PKC and L-selectin shedding). These previous biochemical findings, together with work from this current study, provide compelling evidence for divergent roles of ezrin and moesin in regulating L-selectin-dependent signalling and ectodomain shedding, respectively, during TEM. Interestingly, evidence from moesin-null mice reveal that CD4 and CD8 single-positive T-cells have significantly higher levels of L-selectin on their surface (Hirata et al., 2012). This observation is not phenocopied in ezrin-deficient T-cells (Shaffer et al., 2010), again implying that moesin may act as the pro-shedding factor for L-selectin *in vivo*.

By using TA and TD mutants of moesin–GFP, we were able to show that TD cells formed significantly fewer subendothelial pseudopods during TEM than WT cells, but TA cells did not (Fig. 2C,D). This finding is unexpected, as we would anticipate that cycling between phosphorylated and non-phosphorylated forms of moesin is likely to play a fundamental role in regulating pseudopod protrusion during TEM. It is possible that the endogenous ezrin could have a more dominant role in compensating for TA rather than TD moesin in pseudopod protrusion during TEM. Of note, epidermal growth factor stimulation of human A-431 dermal epithelial cells can cause the translocation of cytosolic TA moesin into membrane structures such as ruffles or microvilli (Yonemura et al., 2002). This observation underscores the importance of how PIP_2_ binding can override the need for T558 phosphorylation to activate moesin. Although the activation status of TA moesin–GFP has not been formally tested in THP-1 cells, this result does suggest that TA moesin may play an active role in driving pseudopod formation during TEM.

We show that blocking moesin PIP_2_ binding significantly reduces T558 phosphorylation and thus activation of moesin in monocytes (Fig. 1H,I). Although this data supports similar observations in other cell types (Fievet et al., 2004; Hao et al., 2009; Hirao et al., 1996; Yonemura et al., 2002), this is the first example described in leukocytes undergoing TEM. Enhancing phospholipase C activity can dramatically reduce the bioavailability of PIP_2_ in leukocyte membranes and disable the cross-linking activity of even constitutively activated (TD) ERM proteins (Hao et al., 2009). Pseudophosphorylation of ezrin at threonine 567 (through introduction of a T567E mutation) renders ezrin constitutively active *in vivo*. However, the T567E protein bypasses the requirement for PIP_2_-mediated activation. By analysing cells captured in mid-TEM, we have shown that active ERM proteins cannot pass readily from uropod membranes to pseudopod membranes without undergoing a cycle of C-terminal dephosphorylation. *In vivo*, T567E T-cells present significantly higher cortical tension and delayed TEM rates (Liu et al., 2012). As T567E T-cells can, in time, undergo successful TEM, it is likely that the dominant effect of T567E ezrin may be overridden by compensatory mechanisms. For example, a reduction in the bioavailability of PIP_2_ (e.g. through enhanced phospholipase C activity), could release constitutively active ezrin from membranes. Although not yet proven, it is likely that ERM protein inactivation during TEM could provide a window of opportunity for L-selectin to switch binding partners from ezrin to moesin.

Mechanistically, it is not clear how L-selectin switches affiliation from ezrin to moesin during TEM. Recent biophysical evidence suggests that the tail of L-selectin can interact with phosphatidylserine (PS) residues enriched within the inner leaflet of the plasma membrane, obscuring sites for binding partners such as calmodulin. *In vitro*, moesin can desorb the L-selectin tail from PS-rich membranes, which in turn allows the extended form of calmodulin to bind (Deng et al., 2013a,b). Given the high level of conservation in structure and amino acid identity between the FERM domains of ezrin and moesin, it is unlikely that this desorbing behaviour is specific to moesin. Another possibility is that one of two serine residues in the human L-selectin tail (N-RRLKKGKKSKRSMNDPY-C) is phosphorylated, which may regulate the binding of ezrin and moesin during TEM. Indeed, we have previously shown that mutating serine 364 to alanine (S364A) increased binding between calmodulin and L-selectin; in contrast, mutating serine 367 to alanine (S367A) reduced interaction with calmodulin (Rzeniewicz et al., 2015). We concluded that the S367A mutant could interact more favourably with PS-enriched membranes, reducing overall binding to calmodulin. This would suggest that phosphorylation of S367 could act as part of the desorbing mechanism, whereas phosphorylation S364 could act to regulate the interaction between ERM proteins and calmodulin. Indeed, the FRET efficiency between ezrin and WT L-selectin was consistently higher than with ΔM-N L-selectin (Fig. 7A,D). It is possible that the homeostatic regulation of L-selectin serine phosphorylation is profoundly altered when shedding is blocked. A future focus of our work will be to determine how serine phosphorylation of the L-selectin tail may affect its binding to ERM proteins and in turn regulate L-selectin clustering during TEM.

## MATERIALS AND METHODS

### Antibodies and reagents

Unless otherwise stated, all reagents were purchased from Sigma-Aldrich. Generation of the anti-human L-selectin monoclonal antibody (IgG1) LAM1-14 is described and validated in previous reports (Rzeniewicz et al., 2015; Spertini et al., 1991) and is a kind gift from Thomas F. Tedder (Duke University, Durham, NC). Rabbit anti-moesin (Q480), anti-ezrin (3145S), anti-phospho-ERM (3726) anti-VE-cadherin (D87F2) antibodies were purchased from Cell Signaling Technology. Sheep anti-PECAM-1 (AF806) antibody was purchased from R&D Systems. Phalloidin conjugated to Alexa Fluor 633 and all Alexa Fluor-conjugated secondary antibodies were purchased from Life Technologies. The anti-L-selectin antibody DREG56 was purified from supernatants of a hybridoma cell line (ATCC^®^ HB­300^TM^) and used in flow cytometry experiments (Fig. 8A). All antibodies were used at a 1:400 dilution and phalloidin was diluted 1:300.

### Cell lines and culture

All cell lines were cultured at 37°C in medium containing 5% CO_2_ under humidifying conditions. The THP-1 monocytic cell line was purchased from the American Type Culture Collection (LGC Standards) and passaged in RPMI medium containing 10% fetal calf serum (FCS), 1% antibiotics (penicillin/streptomycin) and 50 μM β-mercaptoethanol. Cells were tested negative for mycoplasma. HUVECs were purchased from Lonza and maintained in endothelial cell growth medium (EGM-2) supplemented with growth factors and antibiotics provided within their ‘bullet kits’. Cells were initially expanded for six or fewer passages, and were harvested and stored in liquid nitrogen for final use in flow assays or western blotting. Confluent HUVECs were disaggregated with trypsin/EDTA solution and seeded onto 10 μg/ml bovine-derived fibronectin. HEK 293T cells were used for lentiviral production and were a kind gift from Yolanda Calle, University of Roehampton, London, UK. THP-1 cells were maintained in growth medium [RPMI-1640 medium supplemented with 10% FCS and 1% antibiotics (penicillin/streptomycin) and 50 µM β-mercaptoethanol]. Cells were routinely passaged at a 1:3 ratio on the third day. This ‘splitting’ activity maintained an optimal cell density at 0.5×10^6^ cells per ml.

### Knockdown of moesin and ezrin in THP-1 monocytes

Moesin shRNA lentiviral particles were purchased from Sigma-Aldrich (Mission^®^ technology range). The target sequences in moesin are listed below, and they reside either in the 3′ untranslated region (3′ UTR) or the coding domain sequence (CDS). Lentiviral clones 1–3 were provided in the pLKO.1-puro vector, whereas clones 4 and 5 were provided in TRC2-pLKO.1-puro vector.

Clones 1 and 4, 5′-CCGGGCTAAATTGAAACCTGGAATTCTCGAGAATTCCAGGTTTCAATTTAGCTTTTTG-3′ (3′ UTR); clone 2, 5′-CCGGGCATTGACGAATTTGAGTCTACTCGAGTAGACTCAAATTCGTCAATGCTTTTTG-3′ (CDS); clone 3, 5′-CCGGGCGGATTAACAAGCGGATCTTCTCGAGAAGATCCGCTTGTTAATCCGCTTTTTG-3′ (CDS); clone 5, 5′-CCGGACCACCGGGAAGCAGCTATTTCTCGAGAAATAGCTGCTTCCCGGTGGTTTTTTG-3′ (CDS).

Cells were selected using 1 µg/ml of puromycin in THP-1 medium and passaged to maintain cultures at densities of 0.5×10^6^ cells per ml. Western blotting confirmed knockdown efficiency against THP-1 cells expressing non-target (NT) control shRNA, which was also supplied as lentiviral particles by Sigma-Aldrich.

Ezrin shRNA lentiviral particles were purchased from Sigma-Aldrich and targeted the following sequences: clone 3, 5′-GTACCGGTGATGCCCTTGGACTGAATATCTCGAGATATTCAGTCCAAGGGCATCATTTTTTG-3′; clone 4, 5′-GTACCGGGGGCAACCATGAGTTGTATATCTCGAGATATACAACTCATGGTTGCCCTTTTTTG-3′; clone 5, 5′-CCGGCCCACGTCTGAGAATCAACAACTCGAGTTGTTGATTCTCAGACGTGGGTTTTTG-3′; clone 6, 5′-CCGGCGTGGGATGCTCAAAGATAATCTCGAGATTATCTTTGAGCATCCCACGTTTTTG-3′; clone 9, 5′-CCGGCCAGCCAAATACAACTGGAAACTCGAGTTTCCAGTTGTATTTGGCTGGTTTTTG-3′.

### Cloning of lentiviral expression vectors for L-selectin- and ERM proteins-GFP/RFP

Human L-selectin-GFP was generated as previously described (Rzeniewicz et al., 2015). The open reading frames (ORFs) of mouse moesin and human ezrin were subcloned into the pHR'SIN-SEW lentiviral vector containing the ORFs of green or red fluorescent protein (GFP or RFP) 3′ to the multiple cloning site. Each ORF contained unique restriction sites for directional subcloning (moesin, 5′ XhoI–KpnI 3′; ezrin, 5′ XhoI–SalI 3′). All proteins were expressed with C-terminal tags of GFP or RFP. A multi-site QuikChange™ (Stratagene) PCR *in vitro* site-directed mutagenesis protocol was employed in order to introduce mutations using the lentiviral plasmid containing WT moesin–GFP or –RFP.

N-terminal and C-terminal mutations were engineered within cloned lentiviral vectors as follows: 4N, T558A, T558D and 4N-T558D. For single changes of amino acid codons, an alternative protocol was employed using the DNA polymerase Herculase II (Agilent Technologies, USA). The primers used for site-directed PCR mutagenesis are: TA moesin fwd 5′-TGCGACTGGGACGAGACAAATACAAGGCCCTGCGCCAGATCCGGCAGGGCAAC-3′ and rev 5′-ACGCTGACCCTGCTCTCTTTATGTTCCGGGACGCGGTCTAGGCCGTCCCGTTG-3′; TD moesin fwd 5′-TGCGACTGGGACGAGACAAATACAAGGACCTGCGCCAGATCCGGCAGGGCAAC-3′ and rev 5′-ACGCTGACCCTGCTCTCTTTATGTTCCTGGACGCGGTCTAGGCCGTCCCGTTG-3′; 4N moesin fwd 5′-GAATATCTCTTTCAATGATAACAATTTTGTCATCAAGCCCATTGACAATAACGCCCCGGACTTTGTGTTC-3′ and rev 5′-GAACACAAAGTCCGGGGCGTTATTGTCAATGGGCTTGATGACAAAATTGTTATCATTGAAAGAGATATTC-3′.

Sanger sequencing by Source BioScience (Nottingham, UK) ensured correct insertion of desired mutations, and that no spontaneous mutations appeared within the ORF during the mutagenesis procedure.

### Moesin and ezrin rescue THP-1 cell lines

The ORF for mouse moesin was used in rescue experiments. The targeted regions of clones 2 and 3 were sufficiently different and lacked the capacity to knockdown exogenously transduced mouse moesin. Human ezrin was used in rescue experiments and was therefore re-engineered. The 27 base pair sequence that encompasses the underlined target sequence for clone 5 in ezrin is written below. The second sequence shows where the silent mutations were designed (in bold italics) showing 35% of the DNA was rendered non-complementary: endogenous ezrin sequence, 5′-CCACGTCTAAGAATCAACAAGCGGATC-3′; mutated (shRNA-resistant) sequence, 5′-CCACGT***T***T***GCGG***ATCAACAAGCGGATC (amino acids 272–280 in human ezrin, to give sequence of 272-PRLRINKRI-280).

### Monitoring threonine phosphorylation of GFP-tagged moesin and ezrin in cells undergoing TEM under ‘static’ conditions

HUVEC monolayers were grown to confluency on fibronectin-coated six-well plastic dishes (10 µg/ml) and stimulated overnight with 10 ng/ml of recombinant human TNF (R&D Systems). THP-1 cells expressing added-back GFP-tagged ezrin or moesin were harvested and resuspended in cell growth medium to a density of 5×10^5^ per ml. 1 ml of cell suspension was added to each well of HUVECs and incubated for 0, 5, 10, 15, 20, 25 and 30 min at 37°C in a cell culture incubator (under humidifying conditions and 5% CO_2_). The ‘unbound’ and ‘bound’ fractions cells were harvested and lysed in 150 μl of 2× protein-loading buffer (PLB). Using these conditions, under epifluorescence microscopy, we observed that ≥80% cells underwent TEM events in THP-1 cells. Cells lysates were passed through a 25-gauge needle attached to a 0.3 ml insulin syringe to shear nuclear DNA (this process was repeated ∼10 times). Samples were subsequently boiled at 95°C for 5 min and 10 µl was resolved on polyacrylamide gels as described directly below. Levels of phospho-moesin–GFP or phospho-ezrin–GFP were detected by western blotting using an anti-phospho-ERM antibody (Cell Signaling Technology). Phospho-ERM levels were quantified using LI-COR Image Studio software (see below) and the loading of sample was normalised against the GFP signal.

### Western blotting

Precast Novex gels (Life Technologies) were used to resolve whole-cell extracts. Gels were transferred to nitrocellulose membranes (Millipore), which were pre-equilibrated in NuPAGE transfer buffer (Invitrogen) containing 10% (v/v) methanol. Transfer was conducted at a constant rate of 25 V for 2 h. Membranes were blocked in 5% milk buffer in Tris-buffered saline (TBS) containing 0.1% NP-40 for at least 1 h at room temperature. Primary antibody against moesin and ezrin were used at 1:1000 dilution in blocking solution and incubated with continuous agitation overnight at 4°C. Membranes were washed in TBS for 5 min and then in TBS containing 0.1% Tween 20 for 5 min before being washed again in TBS. The membrane was blocked again for at least 60 min at room temperature, after which secondary antibody conjugated to near infrared dyes (680 and 800, LI-COR) was added at a dilution of 1:5000 for a further 60 min at room temperature with gentle agitation. Signal detection was performed using an Odyssey blot scanner (LI-COR). Quantification of protein bands was performed using LI-COR Image Studio software.

### Parallel plate flow chamber assays for live-cell imaging

All flow experiments were performed using a flow chamber 35 mm in diameter that was engineered by Glycotech. All perfusion experiments were performed at 1.5 dyn/cm^2^ assisted by a Harvard Apparatus 2000 PHD syringe pump. Perfusion medium consisted of RPMI supplemented with L-glutamine, 10% FCS, 1% penicillin/streptomycin, 50 μM β-mercaptoethanol, and 25 mM HEPES. HUVECs were seeded onto glass coverslips 35 mm in diameter (no. 1 thickness; VWR) precoated with 10 μg ml^−1^ fibronectin. Before each perfusion assay, HUVECs were stimulated overnight (16 h) with 10 ng ml^−1^ carrier-free recombinant human TNF (R&D Systems). THP-1 cells were perfused at a density of 0.5×10^6^ cells per ml, and primary CD14^+^ monocytes were perfused at a density of 1×10^6^ cells per ml. All flow assays were conducted using an inverted Olympus IX-81 epifluorescence microscope, housed in a Perspex environmental chamber and maintained at a stable temperature of 37°C. An Olympus 10× objective lens (NA=0.3) was used to capture all flow experiments.

### Timed perfusion assays

Under situations where cell protrusion was analysed at 6 or 25 min (i.e. Figs 3, 6 and 7), THP-1 cells were resuspended in perfusion medium (see above) at a density of 2×10^6^ cells per ml. Following 2 min of perfusion of a 1 ml bolus of cells, the tubing carrying the cell suspension towards the flow chamber was transferred into medium alone. Switching the tubing from cell suspension to medium alone did not impact the flow assay in any way. By 6 min perfusion, very few cells were present in the perfusate and most were bound to the HUVEC monolayer, forming early dynamic protrusions. By 25 min perfusion, the protrusions had stabilised (as in Movies 2–4). These two different time points reflected the early and late phases of TEM. We have previously reported that ectodomain shedding of L-selectin–GFP from THP-1 cells is at a maximum by 25 min (Rzeniewicz et al., 2015). These time points were essential for analysing L-selectin–ERM interaction in pseudopods and uropods during periods when ectodomain shedding of L-selectin is minimal (6 min) and maximal (25 min).

### Sample fixation/staining procedures for conventional microscopy and FRET

For confocal analysis, coverslips were detached from the flow chamber at the end of each flow assay and immediately submerged in 4% (v/v) PFA solution for 10–15 min at room temperature. Cells were washed four to five times in PBS to remove excess PFA and permeabilised for 3 min in ice-cold PBS containing 0.1% (v/v) NP-40 substitute (Fluka). After gently washing off the permeabilisation buffer, coverslips were blocked in 10% FCS containing FcR block (Miltenyi Biotec) overnight at 4°C. Specimens were then labelled with appropriate primary and secondary antibodies at a 1:400 dilution (diluted in similar block solution). Note that a PBS wash, followed by a blocking step, was included between primary and secondary antibody staining. Coverslips were finally washed four to five times in PBS and mounted onto glass slides using fluorescence mounting medium (Dako). DAPI staining preceded mounting at 1:5000 dilution.

For FRET/FLIM analysis (see below), PFA-fixed samples were treated with sodium borohydride to eliminate unreacted aldehydes and reduce auto-fluorescence. Samples were then treated with phalloidin conjugated to Alexa Fluor 633 to ascertain the ‘Top’ and ‘Base’ aspects of mid-transmigrating cells.

### Spinning disk and point scanning confocal microscopy

Samples were imaged using a Nikon Spinning Disk confocal system (Nikon Eclipse TiE equipped with a CSU-X unit and an Andor iXON Ultra 897) at the Wohl Cellular Imaging Centre at King's College London. Images were acquired using a 60× oil immersion objective (NA 1.4). Anti-PECAM-1, anti-L-selectin and anti-ezrin/moesin were labelled with secondary antibodies conjugated to Alexa Fluor 405, 488, 568 dyes, respectively. Alexa Fluor 405, 488 and 568 dyes were excited with a 405 nm diode laser, 488 nm diode laser and 561 nm diode laser, respectively. F-actin was labelled with Alexa Fluor 647 conjugated to phalloidin and excited using a 640 nm diode laser. Images were taken as a series of *Z*-stacks and individual *Z*-slices of monocyte uropods and protruding pseudopods (above and below the endothelium monolayer, respectively) were taken for colocalisation image analysis using Volocity software (Perkin Elmer).

In some cases, an inverted SP5 confocal microscope (Leica Microsystems) was used with a 63× oil immersion objective (NA 1.4 oil immersion). GFP was excited at 488 nm with an argon laser, whereas TRITC–phalloidin and Alexa Fluor 633 were excited with helium-neon lasers at 543/568 nm and 633 nm, respectively. Images were acquired as single *Z*-planes (0.75 μm) or as series of *Z*-stacks.

### Isolation of CD14^+^ primary human monocytes

All blood taking was conducted using healthy volunteers and complied with local research ethics committee approval at King's College London (REMAS study reference: HR­15/16­2214). A maximum of 25 ml of freshly isolated venous blood was overlaid onto 15 ml of neat room temperature Histopaque 1077 (Sigma-Aldrich). Cells were spun at 1450 rpm (454 ***g***) for 30 min without acceleration and break. The interface layer containing peripheral blood mononuclear cells (PBMCs) was retained for further purification according to manufacturer's instruction (Monocyte Isolation Kit II, Miltenyi Biotec). Briefly, the Monocyte Isolation Kit II is an indirect magnetic labelling system for the isolation of ‘untouched’ monocytes from human PBMCs. Biotinylated monoclonal antibodies are supplied in a cocktail that selectively depletes all leukocytes other than CD14^+^ monocytes. After labelling PBMCs with the antibody kit, cells were incubated with streptavidin-conjugated magnetic beads and passed through a magnetised column. All PBMCs, other than CD14^+^ monocytes were retained on the column. Eluted cells were retained and purity was assessed to be at or above 90%. Flow cytometry was used to confirm CD14 positivity and 7-aminoactinomycin D positivity (to test viability).

### FRET and FLIM analysis

FLIM measurement of FRET was performed with a multiphoton microscope system as described previously (Rzeniewicz et al., 2015). A Nikon TE2000E inverted microscope, combined with an in-house scanner and Chameleon Ti:Sapphire ultrafast pulsed multiphoton laser (Coherent Inc.), was used for excitation of GFP (at 890 nm). Fluorescence lifetime imaging capability was provided by time-correlated, single-photon counting electronics (SPC 700; Becker & Hickl). A 40× objective (NA 1.3) was used throughout (CFI60 Plan Fluor; Nikon), and data were acquired at 500±20 nm through a bandpass filter (35-5040; Coherent Inc.). Acquisition times of ∼300 s at low excitation power were used to achieve sufficient photon statistics for fitting, avoiding either pulse pile-up or significant photobleaching. Data were analysed as previously described (Parsons et al., 2008; 2005). The FRET efficiency is related to the molecular separation of donor and acceptor and the fluorescence lifetime of the interacting fraction by:
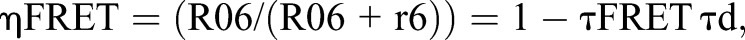


where ηFRET is the FRET efficiency, R0 is the Förster radius, r is the molecular separation, τFRET is the lifetime of the interacting fraction and τd is the lifetime of the donor in the absence of an acceptor. The donor-only control is used as the reference against which all of other lifetimes are calculated in each experiment. τFRET and τd can also be taken to be the lifetime of the interacting fraction and non-interacting fraction, respectively. Quantification of FRET was made from all pixels within each cell that was analysed. All image collection and data analysis were performed using TRI2 software (developed by Paul Barber, Gray Cancer Institute, London, UK).

### Flow cytometry

All flow cytometry (FACS canto, BD Biosciences) and sorting of matched-expressing cell lines (using FACSAria II or MoFlo) were conducted within the Biomedical Research Facility at King's College London.

Cells were labelled with antibody on ice for 30 min, in solution containing Fc Receptor block (Miltenyi Biotec). All washes were performed using 10% (v/v) FCS in Hanks' balanced salt solution (lacking Mg^2+^ and Ca^2+^) with 25 mM HEPES pH 7.4. Cells were subsequently washed and labelled with either DREG56-PE (Santa Cruz Biotechnology, sc-18851) or IgG1-PE isotype control mouse IgG1 (Santa Cruz Biotechnology, sc-2866) both at 1:80 in 50 μl (∼250 ng/10^6^ cells). Cells were washed three times before analysis of protein expression by flow cytometry. A minimum of 20,000 events were counted for each experiment. Data from flow cytometry experiments are represented as the percentage of L-selectin remaining relative to carrier-only treated cells after correction for background fluorescence with isotype-matched control antibodies. All values were normalized against IgG isotype controls.

## Supplementary Material

Supplementary information
