# Supplementary material for: Sequential binding of ezrin and moesin to L-selectin regulates monocyte protrusive behaviour during transendothelial migration
Source: J Cell Sci. 2018 Jul 4;131(13):jcs215541. doi: 10.1242/jcs.215541 (PMC6051341; doi:10.1242/jcs.215541)
Supplement: Supplementary information [file joces-131-215541-s1.pdf]

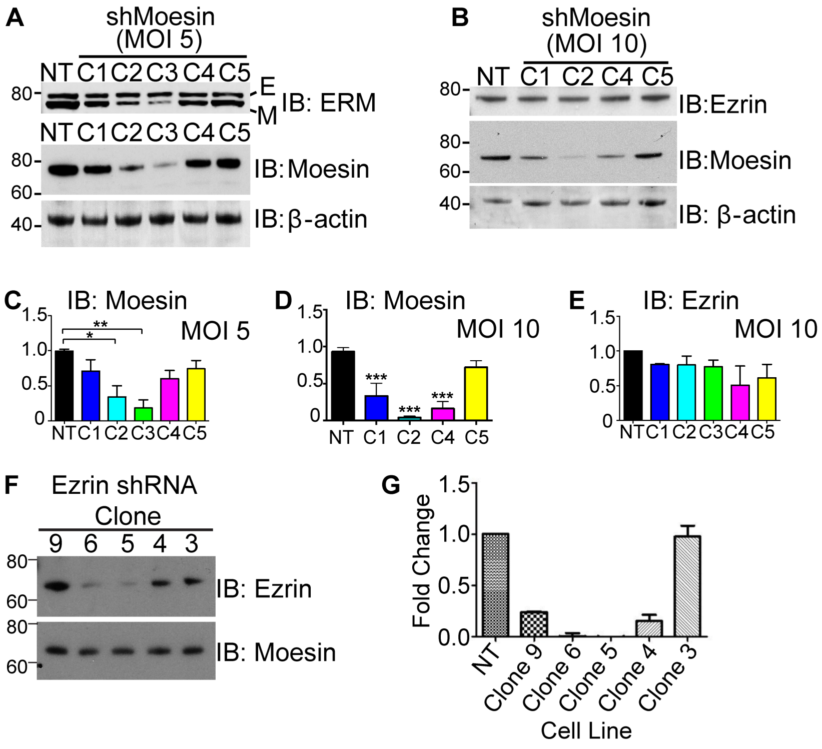

Fig. S1

Systematic knockdown of ezrin and moesin in THP-1 cell lines.

Sigma mission shRNA lentiviral particles, targetting different regions of ezrin and moesin transcripts (termed: clones 1-5 for moesin; clones 3-6 and 9 for ezrin) we in THP-1 cell lines. Full target sequences are listed in Materials and Methods. (**A**) Immunoblots (IB) of whole cell lysates from THP-1 cells were probed with anti-ERM and anti-moesin antibodies. (**B**) Clone 2 at MOI of 10 provided the most stable knockdown and was therefore used in subsequent experiments. (**C**, **D**) Quantification of moesin bands, normalised against β-actin, is representative of three independent experiments. (**E**) Ezrin levels remain unaffected in THP-1 cells treated with Clone 2 shRNA. Relative expression levels of endogenous ezrin are not affected. Values are normalised against cells expressing non-targetting shRNA lentivirus at multiplicity of infection (MOI) 10. (**F**) Ezrin knockdown procedure using Sigma mission lentiviral particles (as in (**A**)). Clones 5 and 6 provided the best knockdown at MOI 5, and quantification of three independent experiments is represented in the graph (**G**). Comparisons are made against non-specific targeted (NT) THP-1 cell lines. One-way ANOVA followed by Bonferroni's post-test. * = $p<0.05$, ** = $p<0.01$, *** = $p<0.001$.

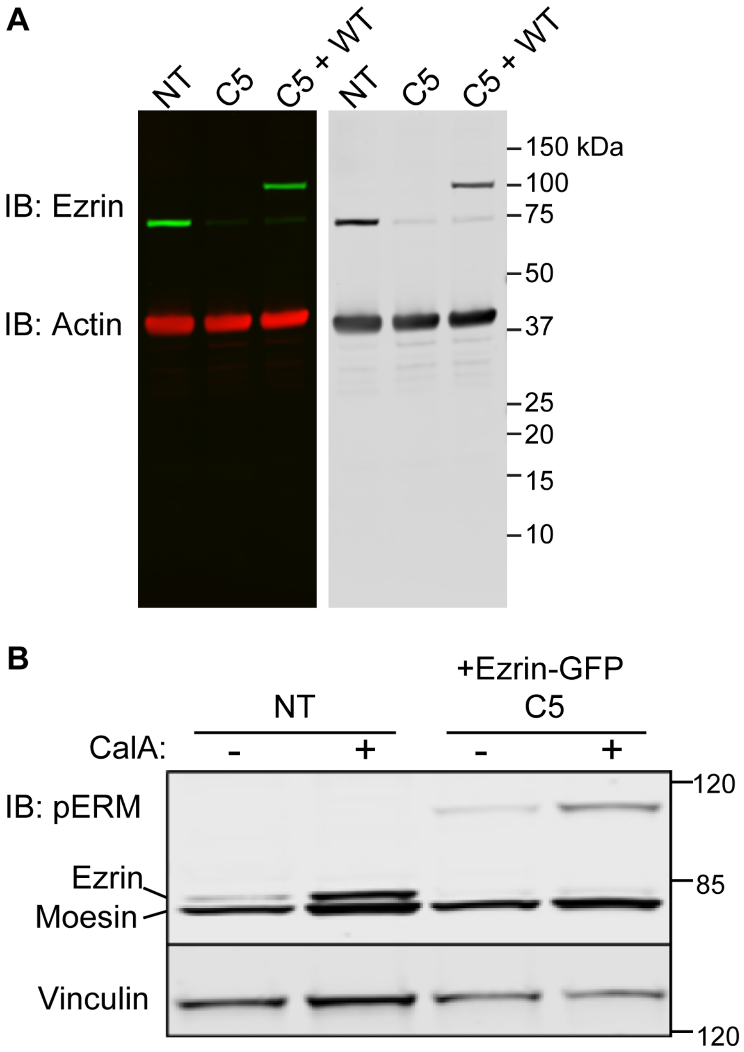

Fig. S2

Reconstitution of ezrin-GFP into knock-down cells.

(**A**) THP-1 cells stably expressing lentiviral shRNA targetting ezrin (clone 5) were reconstituted with shRNA-immune WT ezrin-GFP. Immunoblot shows knockdown of endogenous ezrin and reconstitution of ezrin-GFP to similar expression levels. Actin and ezrin expression were probed simultaneously with anti-ezrin (rabbit, 3145S, Cell Signalling) and anti-actin (mouse monoclonal anti-β-actin, clone AC-74, Sigma Aldrich) antibody. Green and red signals correspond to LI-COR secondary antibody, IRDye® 800CW goat anti-rabbit and IRDye 680LT goat anti-mouse (Odyssey). Merged signals to the right rendered in black and white. Expression levels are compared against THP-1 cells expressing non-targetting (NT) shRNA.

(**B**) Anti-phospho-ERM Western blots reveal that ezrin-GFP can be phosphorylated in response to 25 nM calyculin A (Cal A). THP-1 cells expressing non-targetting shRNA (NT) show the relative phosphorylation levels of ezrin and moesin, which both increase in response to Cal A stimulation. Mouse Anti-vinculin monoclonal antibody (Sigma-Aldrich V9131) was used as loading control in this immunoblot.

Journal of Cell Science • Supplementary information

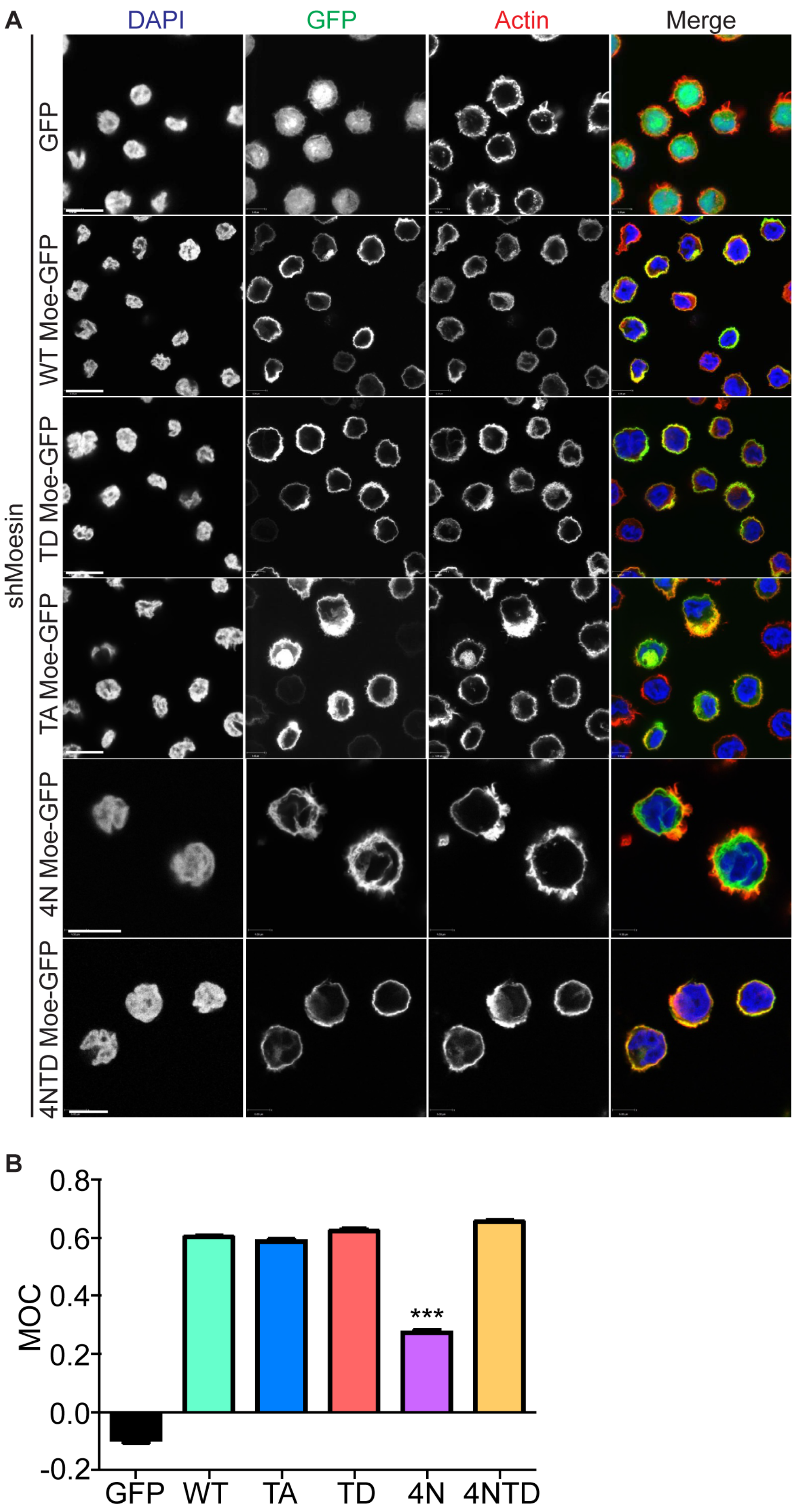

Journal of Cell Science • Supplementary information

Fig. S3

Membrane localisation of WT or mutant moesin-GFP within resting THP-1 cells.

(**A**) THP-1 cells expressing WT or mutant versions of moesin tagged to GFP were harvested and resuspended to a density of 2 x 10^6 cells per mL. Approximately 50 μL of each cell suspension was spotted onto 13 mm diameter poly-L-lysine-coated coverslips and allowed to adhere for 10 min at room temperature. Cells were subsequently fixed in excess (~ 300 μL) 4% paraformaldehyde for 10 minutes, washed in PBS, permeabilised in PBS containing 0.1% (v/v) NP-40 (Fluka) and then blocked in 15% bovine serum albumin for 20 min at room temperature, and stained with TRITC-phalloidin. Panel of images reveal the subcellular localisation of moesin-GFP with TRITC-phalloidin staining. Scale bar for all images = 5.3 μm. TA and TD are respectively constitutively inactive and active forms of moesin (where T558 is mutated to either an alanine (A) or aspartate (D)). The 4N mutant represents the PIP2-binding mutant of moesin, and 4NTD is where the PIP2 binding mutant has been rendered constitutively active.

(**B**) MOC between cells lines reveals significant reduction in membrane localisation of 4N moesin-GFP. Interestingly, the 4NTD mutant associates strongly with phalloidin. This is likely due to the C-terminal domain can interact strongly with the actin cytoskeleton.

Statistical analysis: One-way ANOVA followed by Tukey's post test. ***=p<0.001.

Journal of Cell Science • Supplementary information

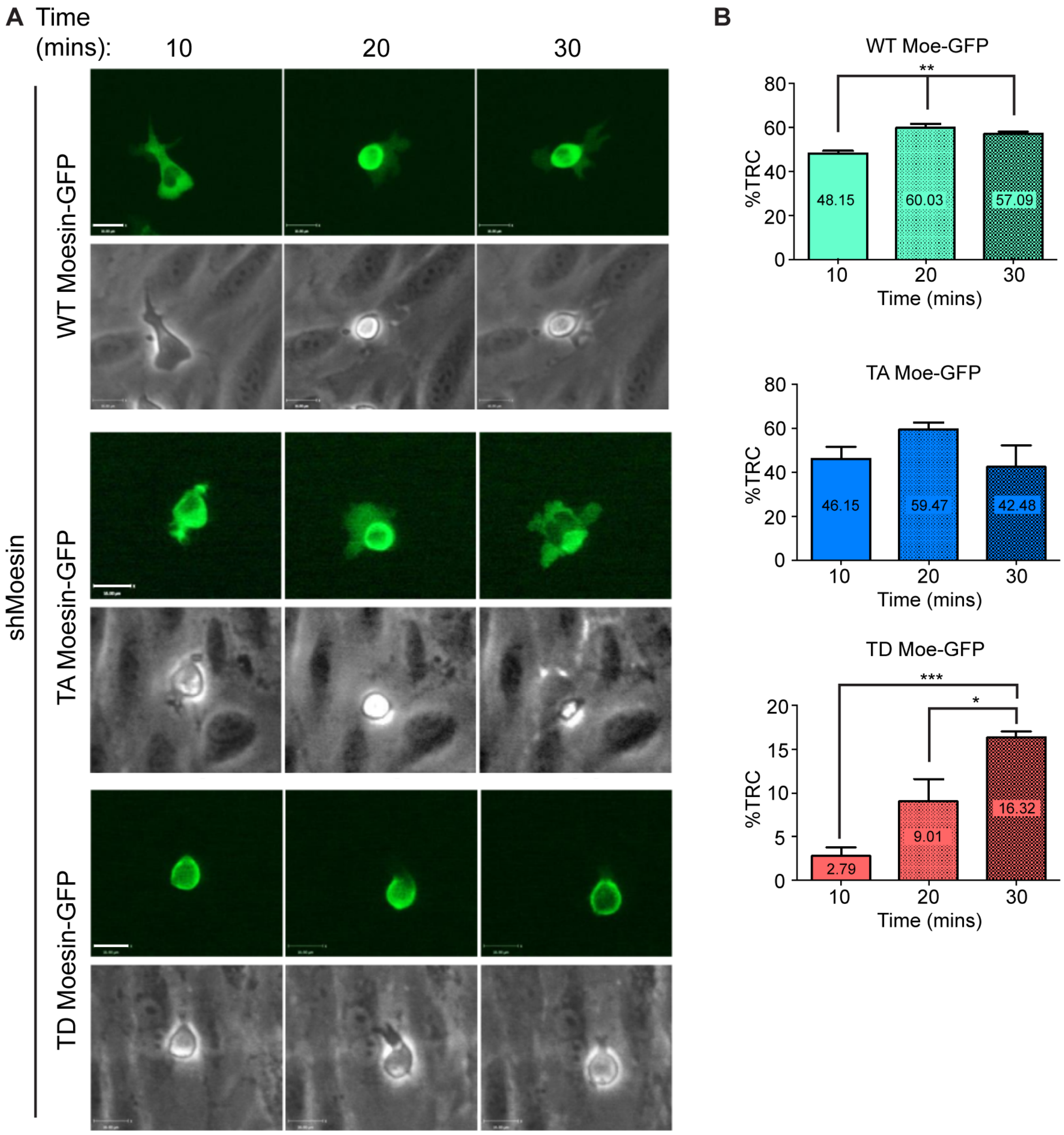

Fig. S4

Monitoring TEM of THP-1 cells expressing WT, TA or TD moesin-GFP.

Endogenous moesin was depleted from THP-1 cells using the clone 2 lentiviral shRNA (Sigma-Aldrich Mission). Cells were subsequently reconstituted with shRNA-resistant WT, TA or TD moesin-GFP to determine to contribution of the C-terminal threonine 558 phosphorylation in regulating TEM (see corresponding Western blots in Fig.1A of main manuscript). Cells were subjected to continuous perfusion for 30 minutes over TNF-activated HUVEC. Timelapse video microscopy allowed THP-1 cells to be scored for forming protrusions during TEM, expressed as a percentage of the total recruited cells (TRC) from flow. (**A**) Three representative images were taken for each cell line: 10 min, 20 min and 30 min. In this example, at 10 min perfusion, cells were found exclusively on top of the endothelium. At 20 and 30 min, only the WT and TA cell lines are forming subendothelial protrusions. Scale bar = 16 μm. (**B**) Data are represented more comprehensively in Figure 2 **C-G** in the main manuscript. One-way ANOVA followed by Bonferroni's post-test. * = $p<0.05$, ** = $p<0.01$, *** = $p<0.001$.

Journal of Cell Science • Supplementary information

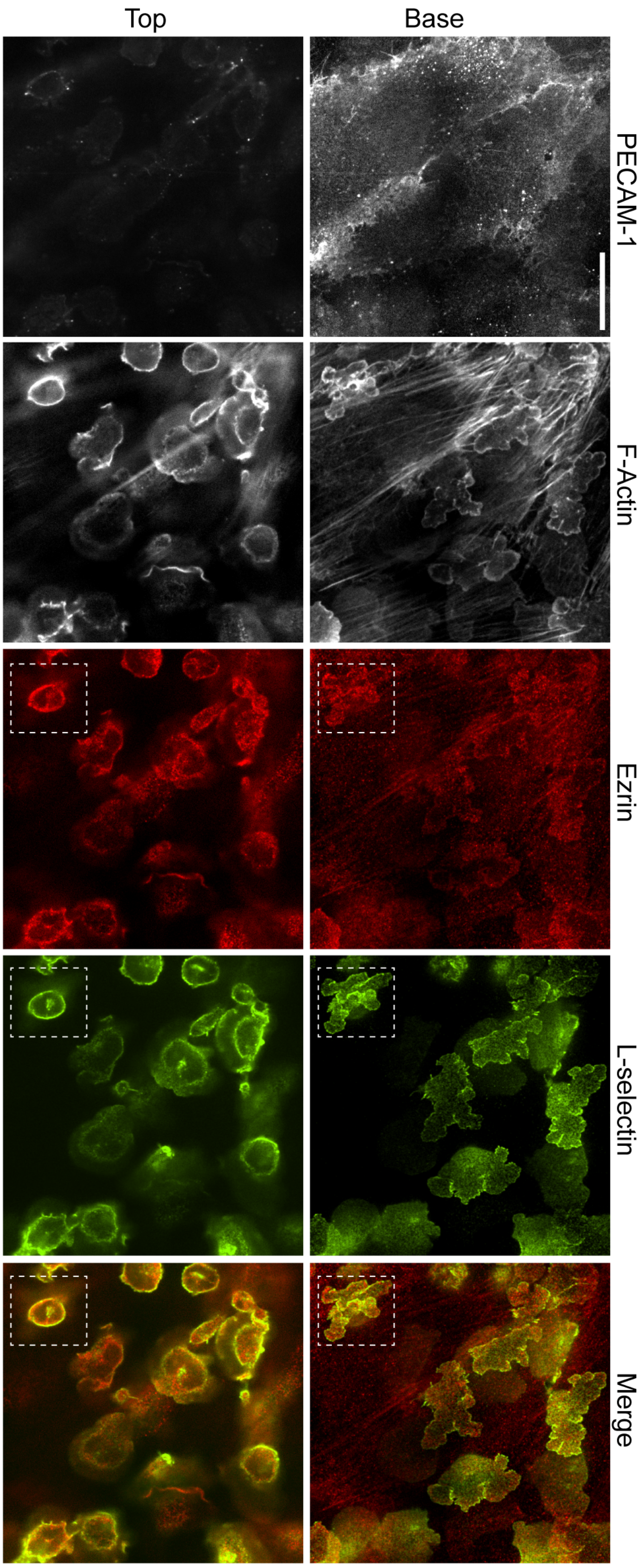

Journal of Cell Science • Supplementary information

Fig. S5

Spinning disk confocal microscopic imaging of CD14-positive primary human monocytes captured in mid-TEM. Monocytes were perfused over TNF-activated HUVEC for 3-6 min and subsequently fixed in 4% PFA. Specimens were prepared for spinning disk confocal microscopy as outlined in materials and methods. Two optical sections capture the non-transmigrated cell body (Top) and transmigrated pseudopods (Base). Dashed box outline identifies the monocyte represented in Figure 4A. PECAM-1 (Alexa Fluor 405 anti-sheep secondary), F-actin (phalloidin Alexa Fluor 633), **Ezrin** (Alexa Fluor 568), and LAM1-14 (Alexa Fluor 488) are all depicted in separate channels. Only signals corresponding to ezrin and L-selectin are merged. Scale bar = 10 μm.

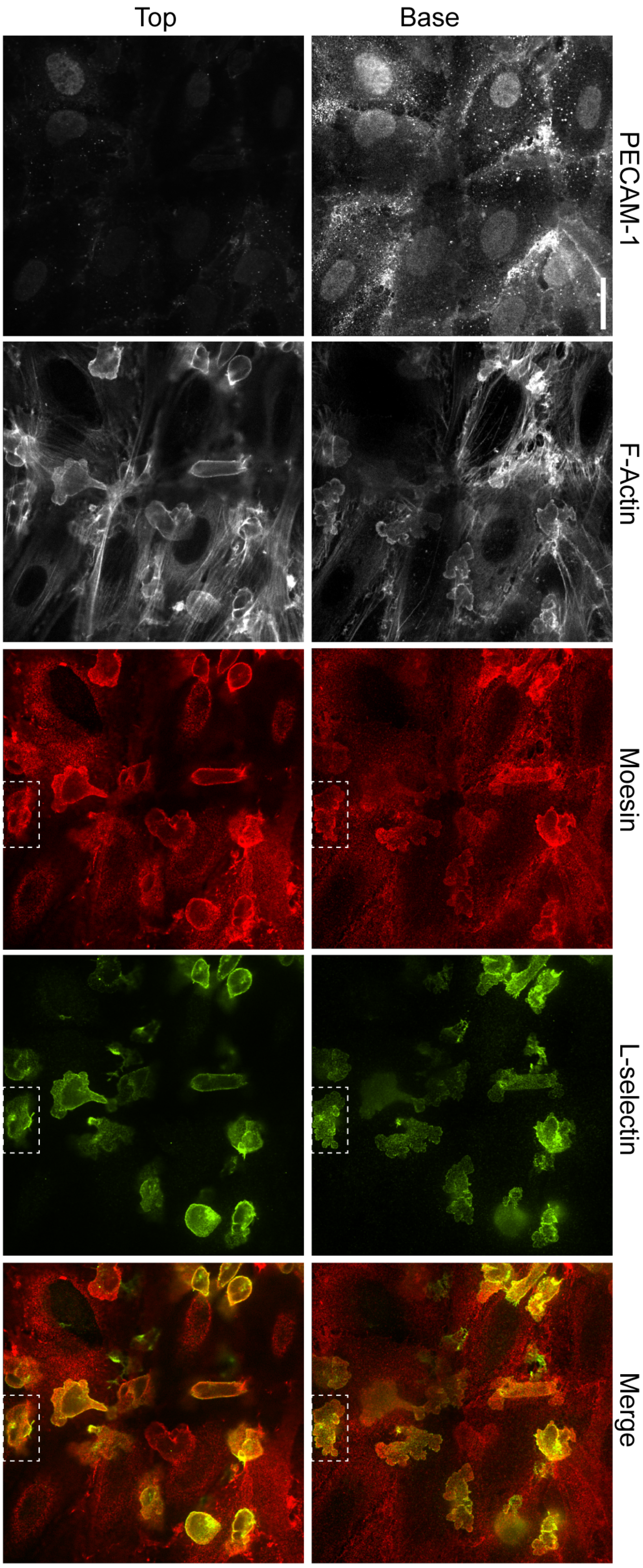

Journal of Cell Science • Supplementary information

Fig. S6

Spinning disk confocal microscopic imaging of CD14-positive primary human monocytes captured in mid-TEM. Monocytes were perfused over TNF-activated HUVEC for 3-6 min and subsequently fixed in 4% PFA. Specimens were prepared for spinning disk confocal microscopy as outlined in materials and methods. Two optical sections capture the non-transmigrated cell body (Top) and transmigrated pseudopods (Base). Dashed box outline identifies the monocyte represented in Figure 4A. PECAM-1 (Alexa Fluor 405 anti-sheep secondary), F-actin (phalloidin Alexa Fluor 633), **Moesin** (Alexa Fluor 568), and LAM1-14 (Alexa Fluor 488) are all depicted in separate channels. Only signals corresponding to moesin and L-selectin are merged. Scale bar = 10 μm.

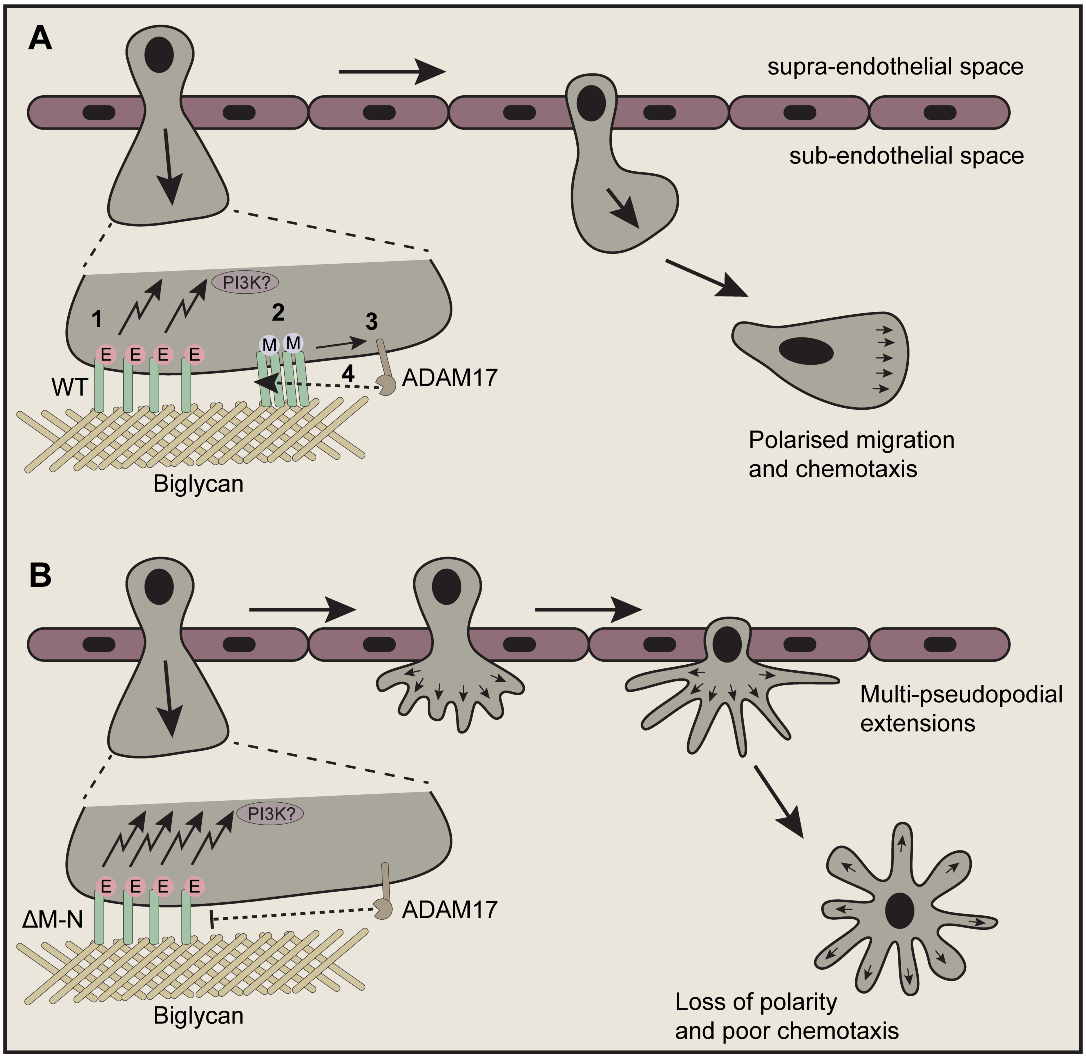

Journal of Cell Science • Supplementary information

Fig S7. Working model: the biological significance underlying sequential interaction of L-selectin with ezrin and then moesin, and its impact on protrusive behaviour during TEM and beyond

(**A**) **Stage 1:** During TEM, the pool of WT L-selectin (green bars) within transmigrating pseudopods interacts preferentially with ezrin (E). Given that ezrin has the potential to interact with the regulatory subunit of PI3K (Gautreau et al., 1999), the biological significance of this interaction could be to promote pseudopod extension during TEM and facilitate cell invasion-like behaviour. **Stage 2:** As time progresses, ezrin is exchanged for moesin (M). In this setting, moesin is likely to contribute to clustering of L-selectin prior to shedding by its protease (ADAM17). **Stage 3&4:** Clustering of L-selectin can drive its own ectodomain shedding in neutrophil suspensions (Palecanda et al., 1992), but whether this can occur specifically in monocytes during TEM is currently not understood. Numerous reports have shown that ectodomain shedding of L-selectin can be triggered by p38 MAPK or PKC (Killock and Ivetic, 2010; Preece et al., 1996; Smolen et al., 2000). Moreover, activation of p38 MAPK lies upstream of ADAM17 mobilisation to the plasma membrane and its proteolytic activity, as judged by threonine phosphorylation of the ADAM17 cytoplasmic tail (Killock and Ivetic, 2010; Xu and Derynck, 2010). L-selectin shedding, specifically during TEM, is essential for the establishment of front-back polarity of transmigrated monocytes.

(**B**) Monocytes that express a non-cleavable mutant of L-selectin (ΔM-N) interact preferentially with ezrin, but not moesin. It is believed that the sustained interaction between ΔM-N and ezrin underlies the excessive multi-pseudopodial phenotype, which could be due to uncontrolled signalling to PI3K. Ultimately, cells will fail to establish front-back polarity, which is essential for interstitial migration towards target sites of inflammation.

Journal of Cell Science • Supplementary information

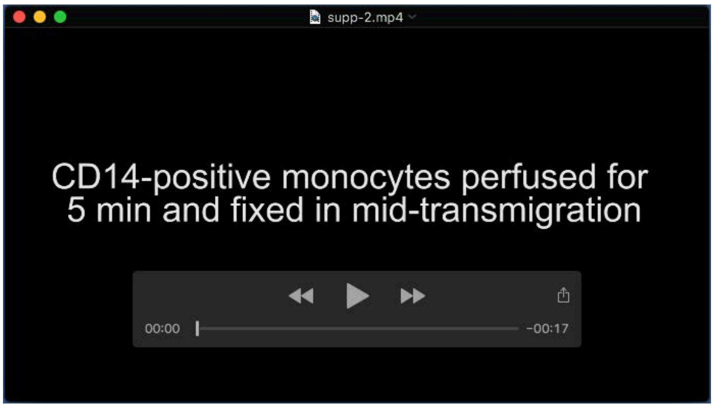

Movie 1
Primary human monocytes were perfused over TNF activated HUVEC for a 4 min and 40 sec at a density of 1.0 x 10^6 per mL. Cells are initially captured from flow, after which they spread, before contracting the spread area just prior to TEM. White arrow shows on the rare occasion a CD14-positive monocyte undergoing full-TEM, highlighting the fact that this flow assay had been optimised for capturing cells in mid-TEM. Scale bar = 100 μm.

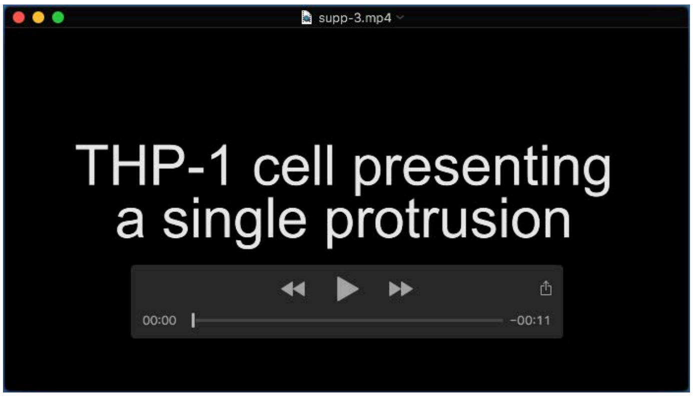

Movie 2
A THP-1 cell stably expressing WT L-selectin-GFP, recruited from flow and subsequently undergoing TEM during the course of the flow assay. Note in this example, the cell is producing a single pseudopodial extension beneath the TNF-activated endothelial monolayer.

Journal of Cell Science • Supplementary information

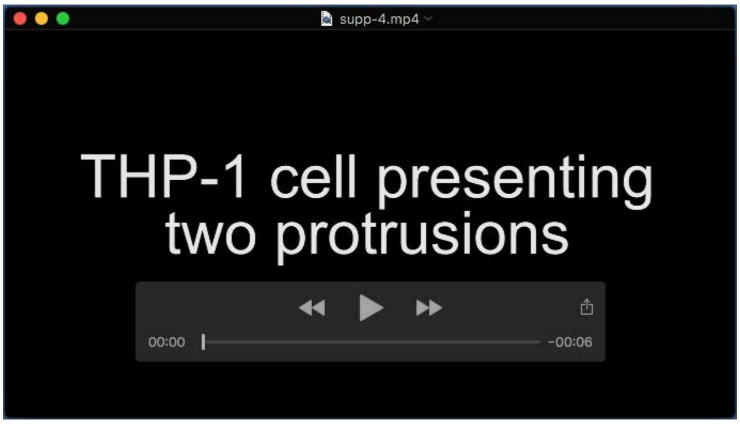

Movie 3
A THP-1 cell stably expressing WT L-selectin-GFP, recruited from flow and subsequently undergoing TEM during the course of the flow assay. The screen is split into phase (left hand side) and GFP (right hand side) channels. Note in this example, the cell is producing two pseudopodial extension beneath the TNF-activated endothelial monolayer.

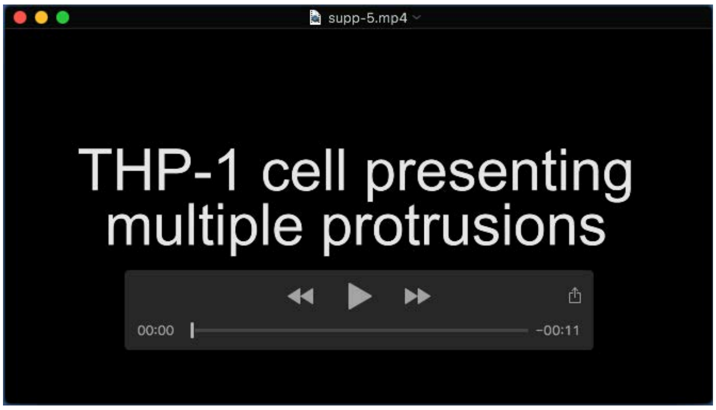

Movie 4
A THP-1 cell stably expressing ΔM-N L-selectin-GFP, recruited from flow and subsequently undergoing TEM during a 25 min period of flow. The screen is split into phase (left hand side) and GFP (right hand side) channels. Note in this example, the cell is producing multi-pseudopodial extensions beneath the TNF-activated endothelial monolayer.

Journal of Cell Science • Supplementary information

SUPPLEMENTAL REFERENCES

Killock, D. J. and Ivetic, A. (2010). The cytoplasmic domains of TNFalpha-converting enzyme (TACE/ADAM17) and L-selectin are regulated differently by p38 MAPK and PKC to promote ectodomain shedding. *Biochem J* **428**, 293-304.

Palecanda, A., Walcheck, B., Bishop, D. K. and Jutila, M. A. (1992). Rapid activation-independent shedding of leukocyte L-selectin induced by cross-linking of the surface antigen. *Eur J Immunol* **22**, 1279-86.

Preece, G., Murphy, G. and Ager, A. (1996). Metalloproteinase-mediated regulation of L-selectin levels on leucocytes. *J Biol Chem* **271**, 11634-40.

Smolen, J. E., Petersen, T. K., Koch, C., O'Keefe, S. J., Hanlon, W. A., Seo, S., Pearson, D., Fossett, M. C. and Simon, S. I. (2000). L-selectin signaling of neutrophil adhesion and degranulation involves p38 mitogen-activated protein kinase. *J Biol Chem* **275**, 15876-84.

Xu, P. and Derynck, R. (2010). Direct activation of TACE-mediated ectodomain shedding by p38 MAP kinase regulates EGF receptor-dependent cell proliferation. *Mol Cell* **37**, 551-66.

Journal of Cell Science • Supplementary information
